# Pulmonary haemorrhage as the earliest sign of severe leptospirosis in hamster model challenged with *Leptospira interrogans* strain HP358

**DOI:** 10.1371/journal.pntd.0010409

**Published:** 2022-05-18

**Authors:** Noraini Philip, Sivan Padma Priya, Ahmad Hussein Jumah Badawi, Mohd Hafidz Mohd Izhar, Norhafizah Mohtarrudin, Tengku Azmi Tengku Ibrahim, Zamberi Sekawi, Vasantha Kumari Neela

**Affiliations:** 1 Department of Medical Microbiology, Faculty of Medicine and Health Sciences, Universiti Putra Malaysia, Serdang, Selangor, Malaysia; 2 RAK College of Dental Sciences, Ras Al Khaimah Medical and Health Sciences University, Ras Al Khaimah, United Arab Emirates; 3 Department of Pathology, Faculty of Medicine and Health Sciences, Universiti Putra Malaysia, Serdang, Selangor, Malaysia; 4 Comparative Medicine and Technology Unit, Institute of Bioscience, Universiti Putra Malaysia, Serdang, Selangor, Malaysia; 5 Department of Veterinary Preclinical Sciences, Faculty of Veterinary Medicine, Universiti Putra Malaysia, Serdang, Selangor, Malaysia; Baylor College of Medicine, UNITED STATES

## Abstract

**Background:**

Severe leptospirosis is challenging as it could evolve rapidly and potentially fatal if appropriate management is not performed. An understanding of the progression and pathophysiology of *Leptospira* infection is important to determine the early changes that could be potentially used to predict the severe occurrence of leptospirosis. This study aimed to understand the kinetics pathogenesis of *Leptospira interrogans* strain HP358 in the hamster model and identify the early parameters that could be used as biomarkers to predict severe leptospirosis.

**Methodology/Principal findings:**

Male Syrian hamsters were infected with *Leptospira interrogans* strain HP358 and euthanized after 24 hours, 3, 4, 5, 6 and 7 days post-infection. Blood, lungs, liver and kidneys were collected for leptospiral detection, haematology, serum biochemistry and differential expression of pro- and anti-inflammatory markers. Macroscopic and microscopic organ damages were investigated. *Leptospira interrogans* strain HP358 was highly pathogenic and killed hamsters within 6–7 days post-infection. Pulmonary haemorrhage and blood vessel congestion in organs were noticed as the earliest pathological changes. The damages in organs and changes in biochemistry value were preceded by changes in haematology and immune gene expression.

**Conclusion/Significance:**

This study deciphered haemorrhage as the earliest manifestation of severe leptospirosis and high levels of IL-1β, CXCL10/IP-10, CCL3/MIP-α, neutrophils and low levels of lymphocytes and platelets serve as a cumulative panel of biomarkers in severe leptospirosis.

## Author summary

As the severe form of leptospirosis could progress rapidly and be potentially fatal if not treated earlier, deciphering the pathophysiology kinetics of infection is crucial to determine the parameters of disease severity. To understand this, we challenged hamsters with the highly virulent *Leptospira interrogans* strain HP358. Pulmonary haemorrhage was observed as the earliest pathological change followed by liver and kidneys damages. The increased expression of IL-1β, CXCL10/IP-10, CCL3/MIP-α, high neutrophils and low lymphocytes and platelets production observed in the present study indicate that these parameters could serve as a cumulative panel of biomarkers in severe leptospirosis.

## Introduction

The severe manifestation of leptospirosis could be either Weil’s disease, a triad of jaundice, renal impairment and haemorrhages; or severe pulmonary forms of leptospirosis (SPFL) without distinct renal and hepatic impairments [1]. The multi-organs involvement often appears as a sudden onset of clinical manifestations, rapidly progressive and associated with high mortality rates [2–5]. This severe manifestation of leptospirosis could be either due to infecting strain of *Leptospira*, the load of leptospiral inoculum and the age and immune status of the infected host.

Despite the existence of the disease for many years, the evolution and factors determining the development of severe leptospirosis in the infected host are still not well defined. Clinical features and pathological changes in severe leptospirosis are described and suggested its association with cytokine storm [6–9]. Several studies have been focused on identifying the factors associated with severe leptospirosis [10–14]. However, the majority of these studies were performed on samples collected at a single time point. For a detailed understanding of the parameters that could be monitored to prevent the illness from progressing to a severe form, a kinetics study of the pathogenesis is vital.

In our earlier study, we isolated and identified a new genotype of *Leptospira interrogans* strain HP358 (*L*. *interrogans* strain HP358) with Sequence Type (ST) 238 in rodents trapped from the hotspot of human leptospirosis in the forest area of Hulu Perdik, Selangor, Malaysia [15]. We performed an *in vivo* pathogenesis screening for the strain HP358 in the hamster model and found that this strain is highly pathogenic manifesting pulmonary haemorrhage, liver and kidneys damages and death as early as six days of post-infection (p.i.) [16]. The evidenced life-threatening clinical manifestations prompted us to investigate and understand the kinetics of the pathophysiology of severe leptospirosis. Therefore, this study was carried out to decipher the progression of the illness by monitoring the clinical manifestation of infected hamsters, histopathological changes in tissues (lungs, liver and kidneys), the leptospiral load, hemogram and serum biochemistry and the cytokines and chemokines expression profiles. We hypothesised that understanding the virulence severity and the time course progression of the disease development may identify factors that are expressed or altered during the early stage of infection which could be recruited for further evaluation and subsequently utilized as biomarkers in severe leptospirosis.

## Methods

### Ethics statement

All experiments were conducted following the guidelines of the Code of Practice for the Care and Use of Animals for Scientific Purposes, Universiti Putra Malaysia. Male golden Syrian hamsters aged between four and six weeks purchased from Monash Universiti Malaysia, Bandar Sunway, Selangor were housed (three per cage) with sterile sawdust bedding, fed with commercial feed and given water *ad lib* in sterile bottles during the study course. The hamsters were acclimatized for 14 days prior to the experiment. All animal procedures carried out in this study were reviewed and approved by the Institutional Animal Care and Use Committee (IACUC), Universiti Putra Malaysia with Animal Use Protocol (AUP) number: UPM/IACUC/AUP-R044/2018. This study also is in compliance with the ARRIVE guidelines.

### Infection, monitoring and euthanization of hamsters

Upon completion of two weeks of acclimatization, the hamsters (n = 21) were infected intraperitoneally (IP) with 2 x 10^8^ of *L*. *interrogans* strain HP358 in 500μl Ellinghausen-McCullough-Johnson-Harris (EMJH) medium. The bacterial load (to develop infection) to be inoculated were selected based on our earlier investigation [16] and in previous studies [17,18]. Control hamsters (n = 7) were injected intraperitoneally with 500μl sterile EMJH medium (without any *Leptospira*). The infectivity study was carried out for seven days. The hamsters were monitored throughout the study for clinical signs such as progressive loss of weight, loss of appetite, reduced physical activity and dyspnea. One control and three infected hamsters were euthanized from day 1 to 7 p.i. except for day 2 to study the pathological events. Due to unforeseen reasons, we were not able to sacrifice the hamsters on day 2. The hamsters were anaesthetized with 100 mg/kg ketamine and 5 mg/kg xylazine injected intraperitoneally and subsequently whole blood was collected by cardiac puncture. Blood was collected for (1) direct culture in EMJH medium, (2) detection of leptospiral DNA and haematological analysis in EDTA tube, (3) biochemistry analysis in plain tube and (4) detection of immune genes in RNAprotect animal blood tube. Hamsters were euthanized by atlanto-occipital dislocation and following dissection, lungs, liver and kidneys were harvested and examined macroscopically for any morphological changes. Twenty-five milligrams of each lung, liver and kidney tissues were collected and transferred into tubes containing absolute ethanol for leptospiral DNA detection and RNAlater for immune genes expression study. The remaining parts of the organs were fixed in 10% neutral buffered formalin for histopathological investigations.

### Macroscopic and microscopic examinations of infected organs

Formalin-fixed organs (lung, liver and kidney) were processed for light microscopy and stained with hematoxylin and eosin (H&E) using the standard protocol. Lesions and changes in the target organs were graded according to previously reported criteria [17,19,20].

### *Leptospira* growth and DNA quantification in blood and organs

Portions of the kidneys of all hamsters were cultured in EMJH medium and observed for up to two months for the growth of leptospires. Leptospiral DNA from blood, lungs, liver and kidneys were extracted using the DNAeasy Blood & Tissue Kit (Qiagen, German) according to the manufacturer’s instructions. The 242bp *lipL32* (primers: LipL32-45F: 5′-AAGCATTACCGCTTGTGGTG-3′, LipL32-286R: 5′-GAACTCCCATTTCAGCGATT-3′, probe: LipL32-189P: FAM-5′-AAAGCCAGGACAAGCGCCG-3′-BHQ1) [21] gene amplification was performed for detection of leptospires in blood and organs. A serial dilution of pure culture of *L*. *interrogans* strain HP358 was used as a standard curve to determine the leptospiral counts, linear range, efficiency and reproducibility of the qPCR assay.

### Haematology and serum biochemistry analyses

Blood samples taken from the hamsters were sent to the Haematology and Biochemistry laboratory, Faculty of Veterinary Medicine, Universiti Putra Malaysia for complete blood counts and biochemical analysis. The parameters for biochemical analysis were selected based on their association with organs damage in human leptospirosis.

### Expression of pro-inflammatory and anti-inflammatory markers

#### Total RNA extraction

Total RNA from blood (RNeasy Protect Animal Blood kit, Qiagen, German), lungs, liver and kidneys (HiYield Total RNA Mini Kit) were extracted following the manufacturers’ instructions. The extracted RNA was eluted in 20μl of RNase-free water. Before storage at -80°C, the quantity and quality of the purified RNA were measured using the NanoDrop 2000 spectrophotometer (Thermo Fisher Scientific) at OD 260/280 and OD 260/230 ratios. The integrity of RNA was verified using gel electrophoresis.

#### Reverse-transcription

DNA-free total RNA extracted from blood, lungs, kidneys, (1μg) and liver (0.5μg) was reverse transcribed into cDNA using the Quantinova Reverse Transcription kit (Qiagen, German). Genomic DNA from the RNA samples was removed using gDNA removal mix (2μl). The total volume of 20μl reverse transcription (RT) reaction mix contained RT enzyme (1μl), RT mix (4μl) and template RNA (entire gDNA elimination reaction, 15μl). RT was conducted on a BioRad machine and consisted of annealing (3 min, 25°C), RT step (10 min, 25°C) and inactivation step (5 min, 85°C). The transcribed cDNA was diluted in 1:2.5 with RNase-free water and kept at -40°C until used.

#### Real-time PCR and amplification program

Primers for immune genes were synthesized (MyTACG Bioscience Enterprise, Malaysia) utilizing sequences from the previous studies (Table 1). These immune genes were selected based on the association of these genes with leptospirosis. For every sample, the amplification (real-time PCR) was carried out in duplicates containing 1μl cDNA in a 20μl final volume for each cytokine and chemokines (Table 1) using Quantinova SYBR green I master kit (Qiagen, German). The amplification was performed on the Eppendorf instrument using Realplex software. The amplification program consisted of an activation step at 95°C for 2 min followed by amplification cycle of the target cDNA for 40 cycles (95°C for 5 s and a combined annealing/extension at 55.7°C for 10 s). Negative control with RNase-free water was included in each run. The specificity of the amplification was verified by analysis of the melting curves of the PCR products.

**Table 1:** List of inflammatory markers and primers used in this study.

No.	Inflammatory markers	Type	Primers sequences	Size bp	References
**Pro-inflammatory**					
1.	Hamster -IFN-γ	Cytokine	F-GACAACCAGGCCATCC R-CAAAACAGCACCGACT	226	[22]
2.	Hamster IL-1β	Cytokine	F-ATCTTCTGTGACTCCTGG R-GGTTTATGTTCTGTCCGT	156	[17]
3.	Hamster IL-6	Cytokine	F-AGACAAAGCCAGAGTCATT R-TCGGTATGCTAAGGCACAG	252	[17]
4.	Hamster TNF-α	Cytokine	F-AACGGCATGTCTCTCAA R-AGTCGGTCACCTTTCT	278	[17]
5.	Hamster CXCL10/IP-10	Chemokine	F-CTCTACTAAGAGCTGGTCC R-CTAACACACTTTAAGGTGGG	150	[17]
6.	Hamster CCL3/MIP-1a	Chemokine	F-CTCCTGCTGCTTCTTCTA R-TGGGTTCCTCACTGACTC	210	[17]
7.	Hamster COX-2	Enzyme	F-CAACTCCCTTGGGTGTGA R-TCCTCGTTTCTGATCTGTCT	173	[17]
8.	Hamster-iNOS	Enzyme	F-CCATTCTACTACTATCAGGTCG R-TCGCCTTGTACTGGTTCAT	274	[23]
**Anti-inflammatory**					
9.	Hamster IL-10	Cytokine	F-TGGACAACATACTACTCACTG R-GATGTCAAATTCATTCATGGC	308	[17]
10.	Hamster TGF-β1	Cytokine	F-ACGGAGAAGAACTGCT R-ACGTAGTACACGATGGG	245	[23]
**Normalization gene**					
11.	Hamster GAPDH	Housekeeping gene	F-CCGAGTATGTTGTGGAGTCTA R-GCTGACAATCTTGAGGGA	170	[17]

#### Gene expression analysis

The level of expression of each gene was normalized to the levels of glyceraldehyde-3-phosphate dehydrogenase (GADPH) housekeeping gene using a comparative delta delta CT method (ΔΔCT Method). The average Ct values of genes tested obtained from the control and infected hamsters for blood and organs (lungs, liver and kidneys) were directly normalized to the reference gene. Then, the difference between the Δct value of infected and control hamsters was calculated to arrive at the double delta Ct value. Finally, the value of 2^-ΔΔCt^ was calculated to obtain the expression fold change. ​

#### Statistical analysis

Statistical analysis was performed using GraphPad Prismv8 (GraphPad Software Inc.) Unpaired t-test was used for the analysis of significant differences in haematology, serum biochemistry and cytokine genes expression between controls and infected animals.

## Results

### Clinical response to infection

The earliest clinical sign observed in the infected hamsters was weight loss which occurred as early as day 3 p.i. Body weight continued to decrease over the days, with average weight loss of 0.3% (D3), 2.2% (D4), 4.6% (D5), 7.3% (D6) and 7.7% (D7). On day 5 p.i., all hamsters showed loss of appetite, reduced physical activity and developed dyspnea. On day 6 p.i., three hamsters died and one hamster died on day 7 p.i.. All control (non-infected) hamsters euthanized on days 1 and 3 to 7 p.i. showed normal behaviour and progressive weight increase (16.6% increase at the end of the study).

### Macroscopically pulmonary haemorrhage occurred earliest

**Fig 1:**
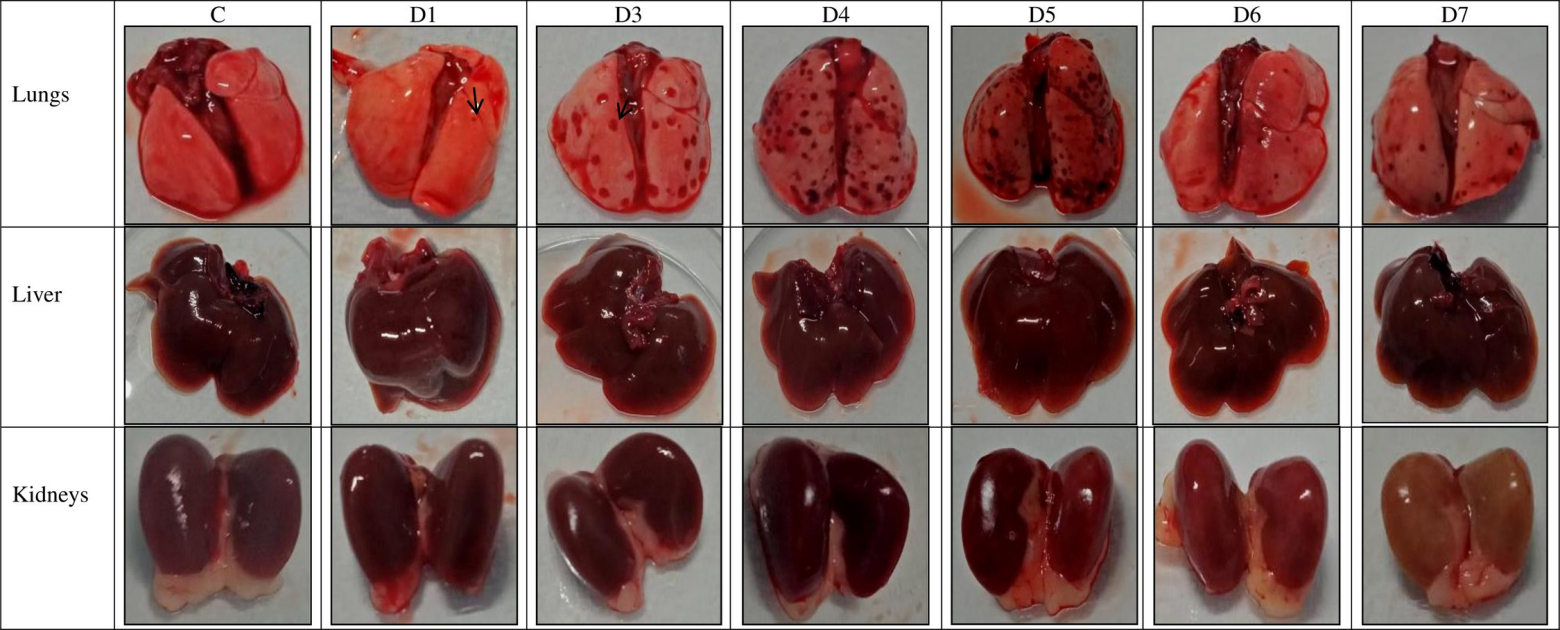
Gross appearance of organs. Petechial haemorrhages were observed in the lungs from day 1 p.i. and became severe from day 3 p.i. onwards. Kidneys became progressively pale from day 6 p.i. onwards. No notable changes were observed in the liver. C: Control; D1-D7: Day 1 to Day 7. The arrow shows a petechial haemorrhage.

**Fig 2:**
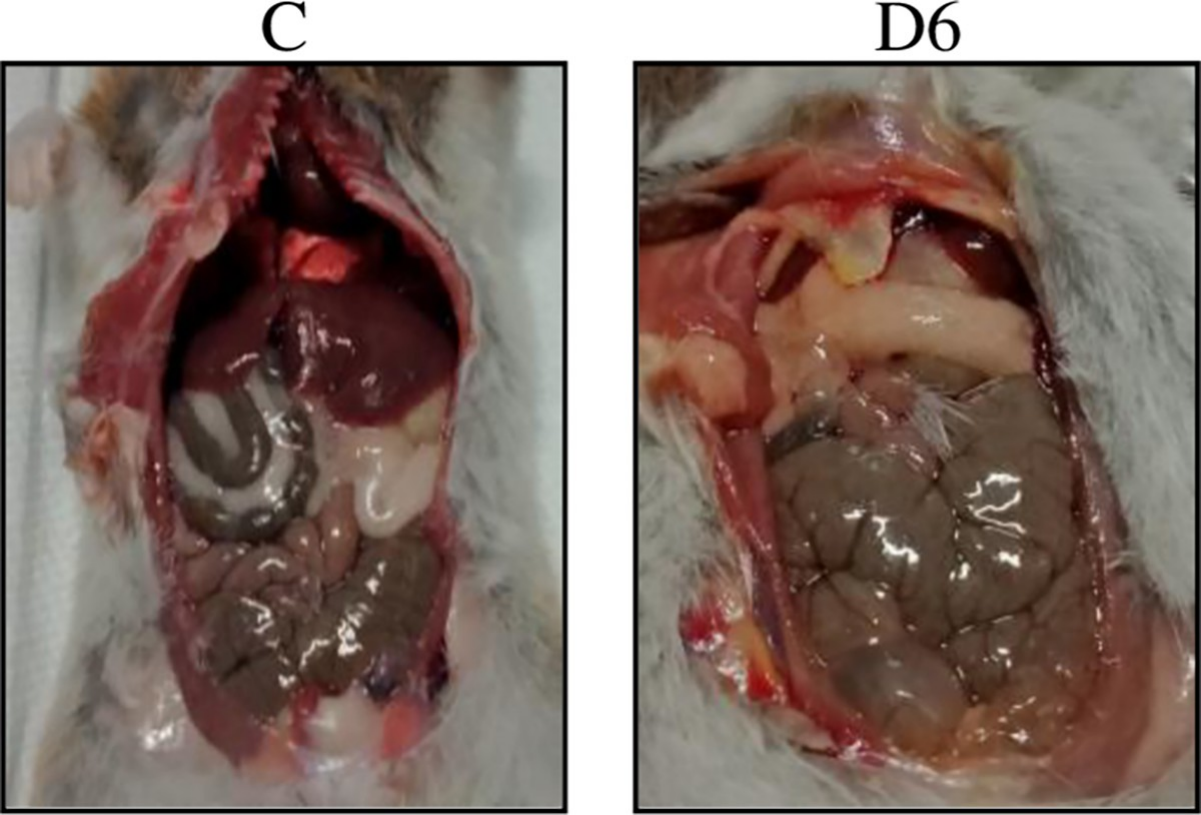
Gross finding in dissected areas. The yellowish discolouration was observed on adipose tissue on day 6 p.i. in infected hamsters. C: control; D6: day 6 p.i.

All three hamsters euthanized on day 1 p.i. showed normal morphology of the liver and kidneys, however, few focal haemorrhagic areas were observed in the lungs. Beginning from day 3 p.i., haemorrhage in the lung continued to spread (Fig 1). The kidneys appeared pale from day 6 p.i. while the liver did not show any marked changes. Another notable finding observed was yellowish discolouration of adipose tissues in some of the dead and euthanized hamsters on days 6 and 7 p.i. (Fig 2). The lungs, liver and kidneys harvested from the control hamsters showed no gross changes.

### Microscopically all organs showed progressive damages

**Fig 3:**
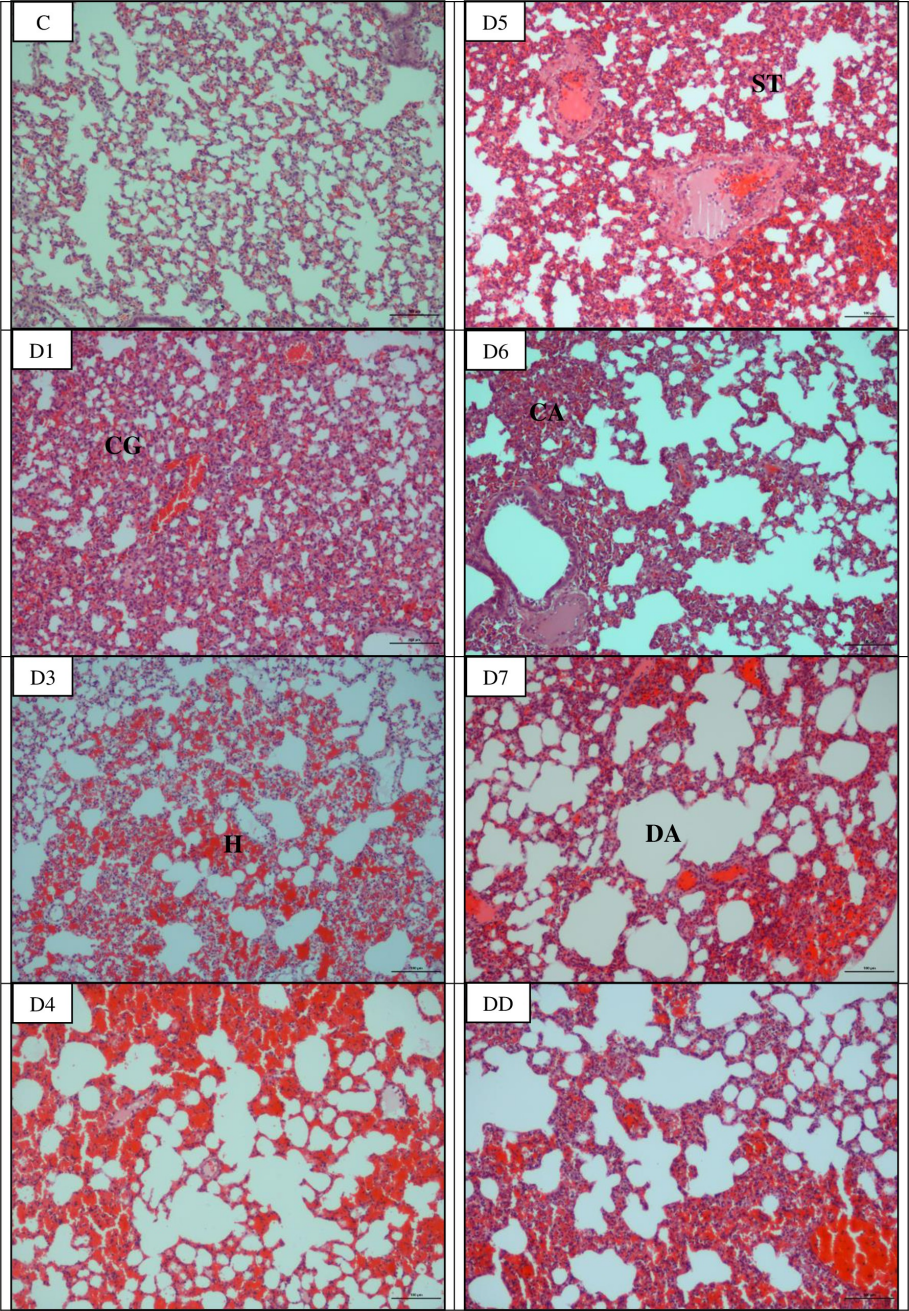
Histopathological lesions in the lung of infected hamsters. Congestion and haemorrhage occurred as early as day 1 p.i. while dilated and collapsed alveoli occurred on days 6 and 7 p.i. and in dead hamsters. CG: Congestion, H: Haemorrhage, CA: Collapsed alveoli, ST: Septal thickening, DA: Dilated alveoli. C: Control, D1-D7: Day 1- day 7 and DD: Dead. Magnification: x100, bar: 100 μm.

**Fig 4:**
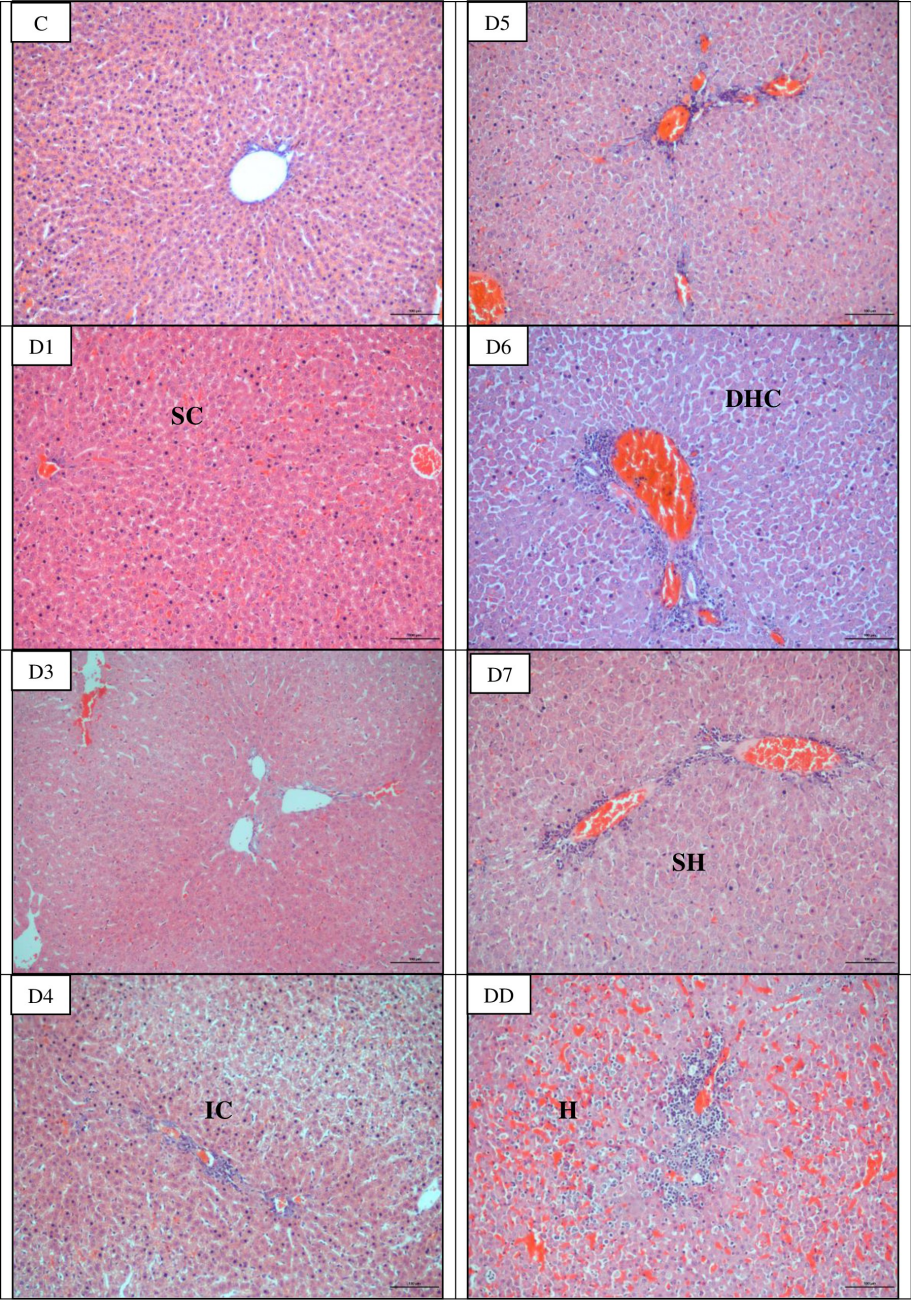
Histopathological lesions in the liver of infected hamsters. Congestion occurred as early as day 1 p.i. while infiltration of inflammatory cells, disorganized hepatocyte cords and swelling of hepatocytes occurred on day 4 p.i. onwards. SC: Sinusoid congestion, IC: Infiltration of inflammatory cells, DHC: Disorganized hepatocyte cords, SH: Swollen hepatocytes and H: Haemorrhage. C: Control, D1-D7: Day 1- day 7 and DD: Dead. Magnification: x100, bar: 100 μm.

**Fig 5:**
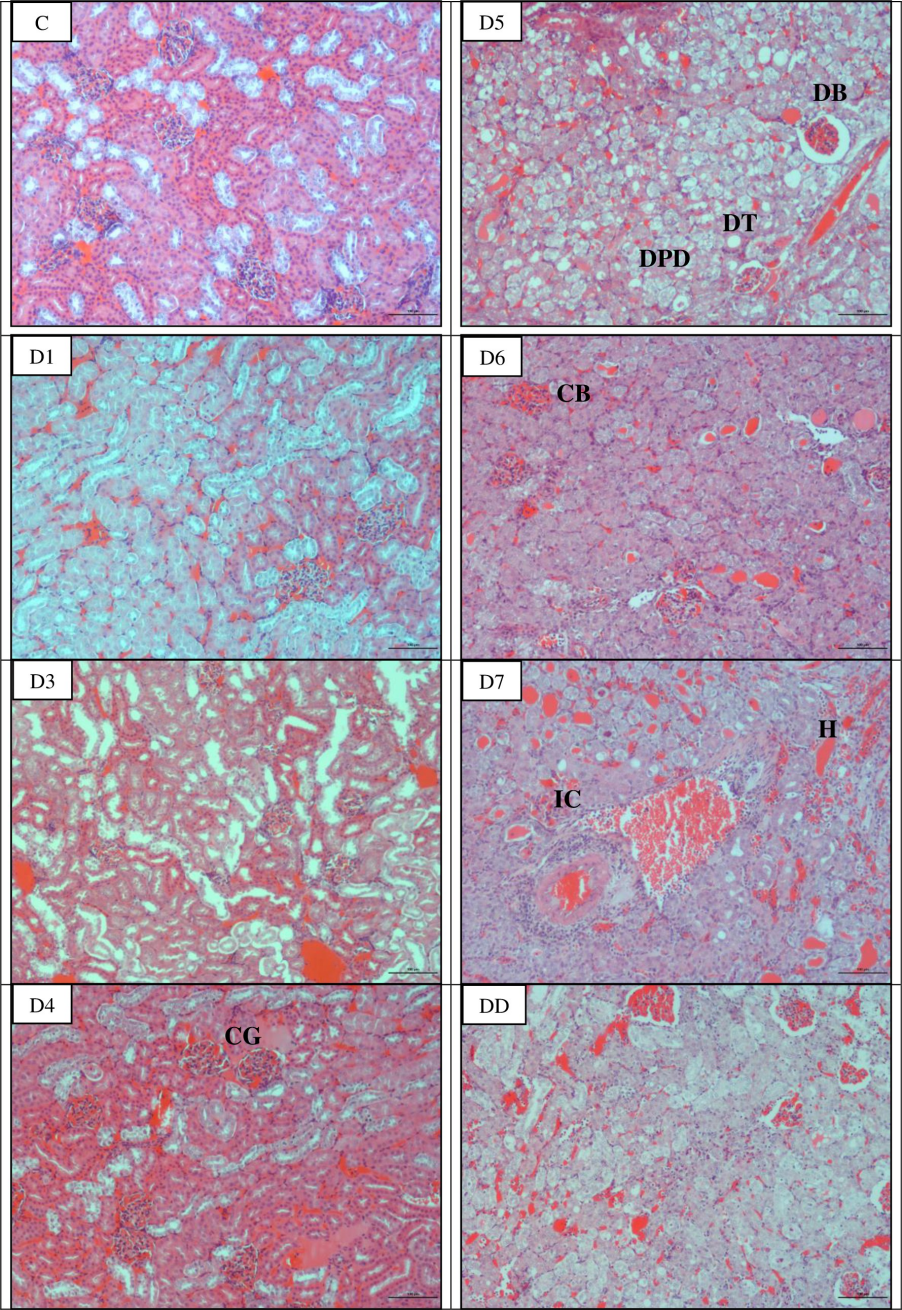
Histopathological lesions in kidneys of infected hamsters. Congestion occurred as early as day 3 p.i., haemorrhage, infiltration of inflammatory cells, dilation and collapse of Bowman’s space on day 4 p.i. and dilation of tubule and degeneration of epithelial cells in proximal and distal tubules occurred on day 5 p.i. onwards. CG: Congested glomerulus, CB: Collapse of Bowman’s space, DB: Dilated Bowman’s space, DT: Dilated tubule, DPD: Degeneration of epithelial cells in proximal and distal tubules, IC: Infiltration of inflammatory cells, and H: Haemorrhage. C: Control, D1-D7: Day 1- day 7 and DD: Dead. Magnification: x100, bar: 100 μm.

The earliest pathological changes observed were congestion of the lung and liver on day 1 p.i. and kidneys on day 3 p.i. (Figs 3–5; Table 2). Haemorrhage was observed in the lung as early as on day 1 and day 4 p.i. in kidneys. In the lung, apart from congestion and haemorrhage, septal thickening and collapsed alveoli were observed from day 1 p.i. while mild alveoli dilation was noted from day 5 p.i. Inflammatory cells infiltration appeared in the liver and kidneys from day 4 p.i. henceforth. In the liver, disorganized hepatic cords and enlargement of hepatocytes were observed from day 4 p.i. and progressively deteriorated. Marked pathological changes were also observed in the kidney. Shrinkage of glomerulus capillaries leading to dilation of Bowman’s space and renal tubular damages characterized by tubular dilation and degeneration of epithelial cells lining of the proximal and distal convoluted tubules were observed in kidneys on day 4 p.i. and henceforth increased in severity. Hamsters that died earlier before day 7 p.i. showed similar pathological changes which were haemorrhagic lungs and kidneys and congested liver. No lesions or any pathological changes were observed in the organs of control hamsters.

**Table 2:** Histological scoring in hamsters infected with *L*. *interrogans* strain HP358.

Parameters	Scoring						
	D1	D3	D4	D5	D6	D7	Dead
**Lungs**							
Capillary congestion	4	4	4	4	4	4	4
Haemorrhage	1	3	4	4	3	3	3
Thickening of interalveolar septa	4	4	4	4	4	4	4
Dilated alveoli (Emphysema)	0	0	0	1	1	1	2
Collapsed alveoli (Atelectasis)	0	0	0	4	4	4	4
**Liver**							
Sinusoid congestion	3	3	3	4	4	4	4
Infiltrating mononuclear or polymorphonuclear cells/inflammatory cells/Periportal hepatitis	0	0	2	4	4	4	4
Disorganized hepatic cords	0	0	2	4	4	4	4
Swelling of hepatocytes	0	0	1	4	4	4	4
**Kidneys**							
Congestion in glomerulus	0	2	4	4	4	4	4
Haemorrhage	0	0	2	4	4	4	4
Dilation of Bowman’s space	0	0	2	2	2	3	4
Collapse of Bowman’s space	0	0	2	2	2	3	4
Dilation of tubule	0	0	0	1	1	1	1
Inflammatory infiltration	0	0	1	2	3	3	3
Degeneration of epithelial cells lining of the proximal and distal convoluted tubules	0	0	0	2	4	4	4

*Scoring system:

0: No lesion

1: Lesion observed in one quadrant

2: Lesion observed in two quadrants

3: Lesion observed in three quadrants

4: Lesion observed in four quadrants/all areas

### Leptospiral load in blood and organs

**Fig 6:**
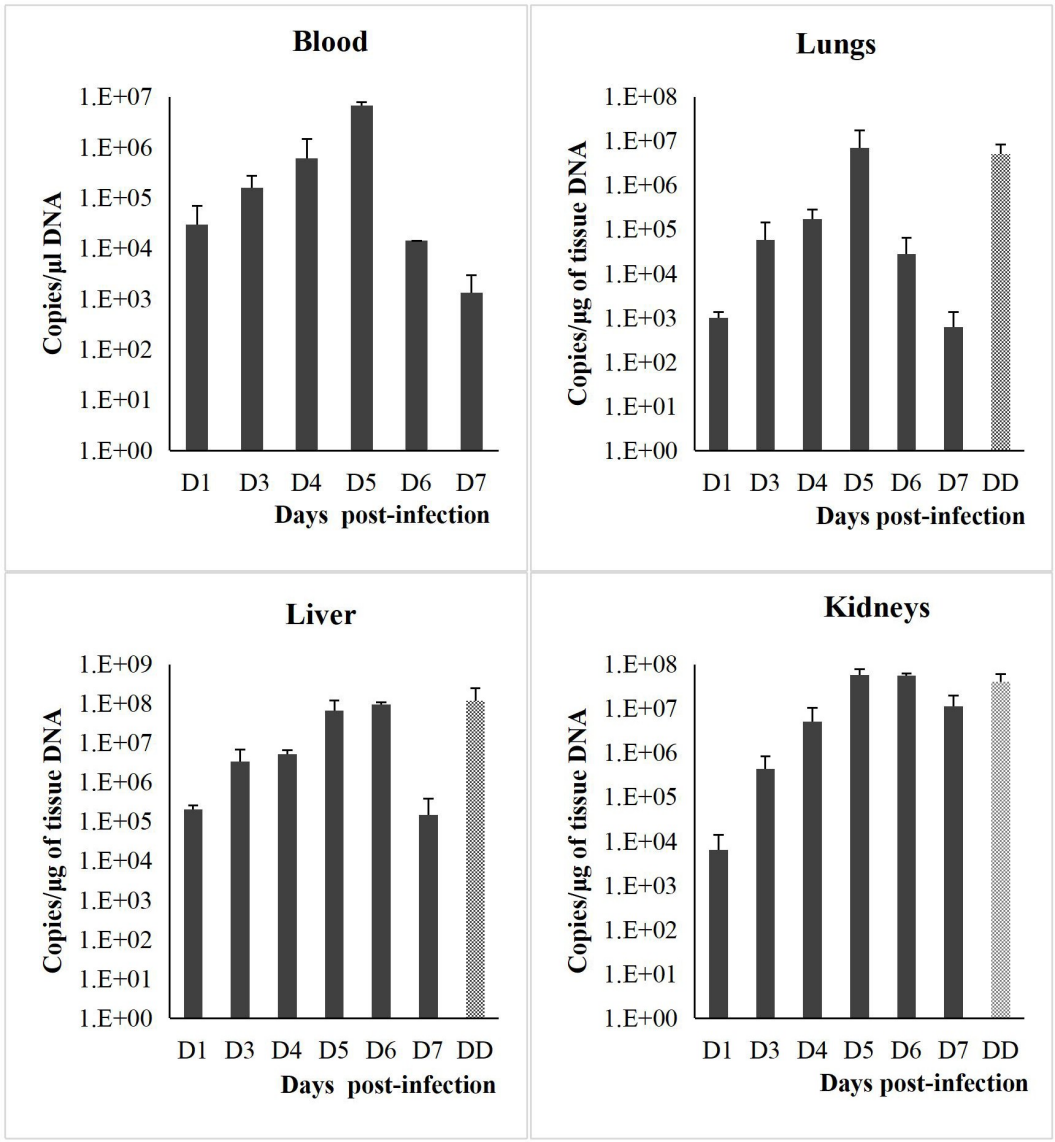
Leptospiral load in blood and organs tissues of hamsters infected with *L*. *interrogans* HP358. D1-D7: day 1 to day 7; DD: dead hamsters. The leptospiral copies number in blood and organs can be found in S1 Table.

Blood and kidneys samples of infected hamsters from day 1 to day 7 p.i. cultured in the EMJH medium yielded positive growth for leptospires while no growth was observed in the control hamsters. Likewise, qPCR also showed positive amplification for all samples (blood, lungs, liver and kidneys) collected from the infected hamsters from day 1 to day 7 p.i. (Fig 6). No cultures were performed for the lungs and liver. The leptospiral load (qPCR) in blood and organs showed a progressive increase from day 1 to day 5 p.i. for blood, lungs and kidneys and until day 6 for the liver. On day 7 p.i., the leptospiral load was lower than day 1 p.i. in blood, lungs and liver while the load remained unchanged in the kidneys.

### Haematological and serum biochemical changes

#### Haematological changes

**Fig 7:**
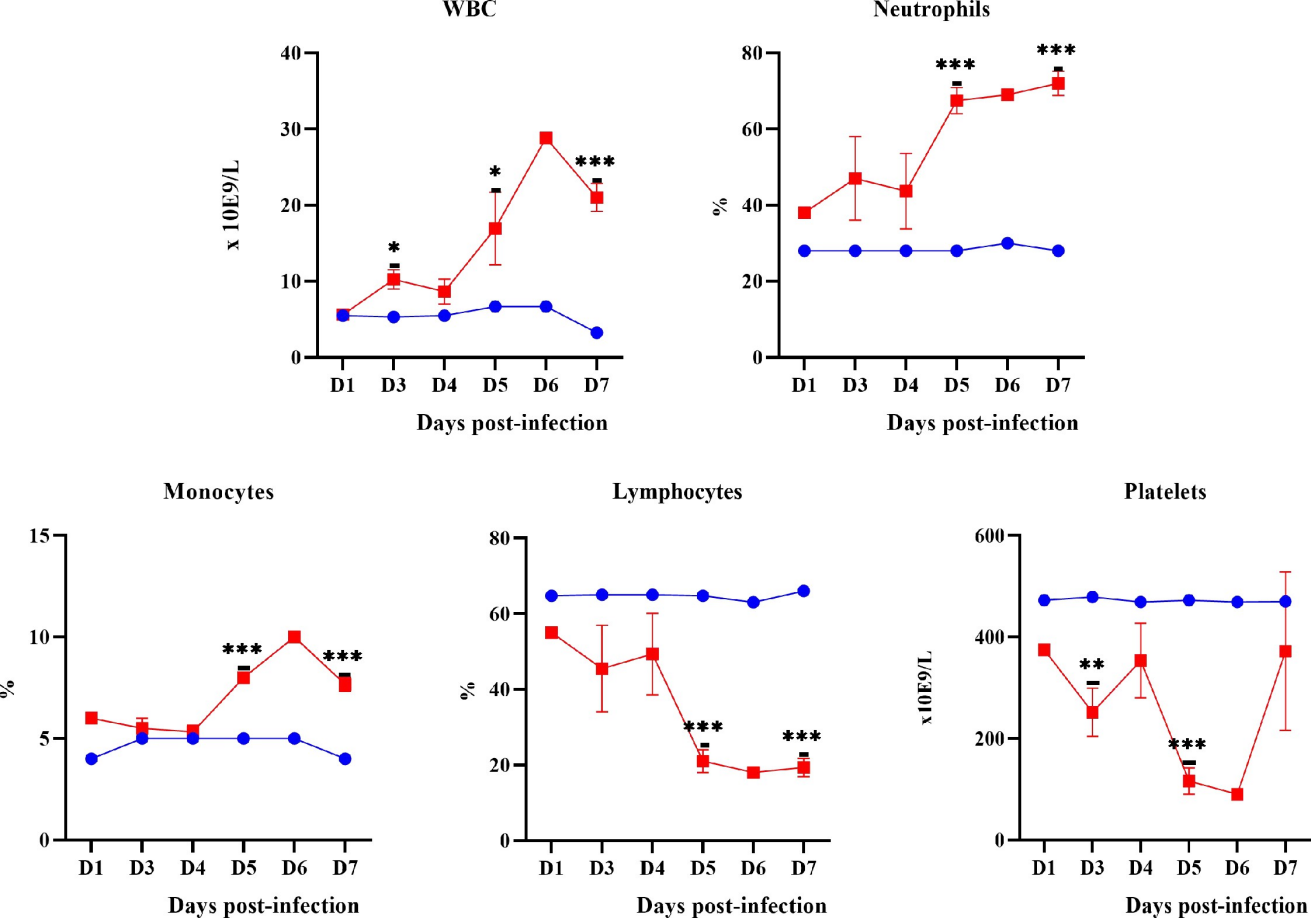
The pattern of WBC, neutrophils, monocytes, lymphocytes and platelets levels in control (blue line) and infected hamsters (red line). D1-D7: Day 1 to day 7. *P≤0.05, **P≤0.01, ***P≤0.001.

White blood cells (WBC), neutrophils and monocytes counts in infected hamsters showed an increase from as early as day 1 p.i. (p-value = <0.05) (Fig 7) while lymphocytes and platelets showed a decreasing trend (p-value = <0.05).

#### Serum Biochemical changes

**Fig 8:**
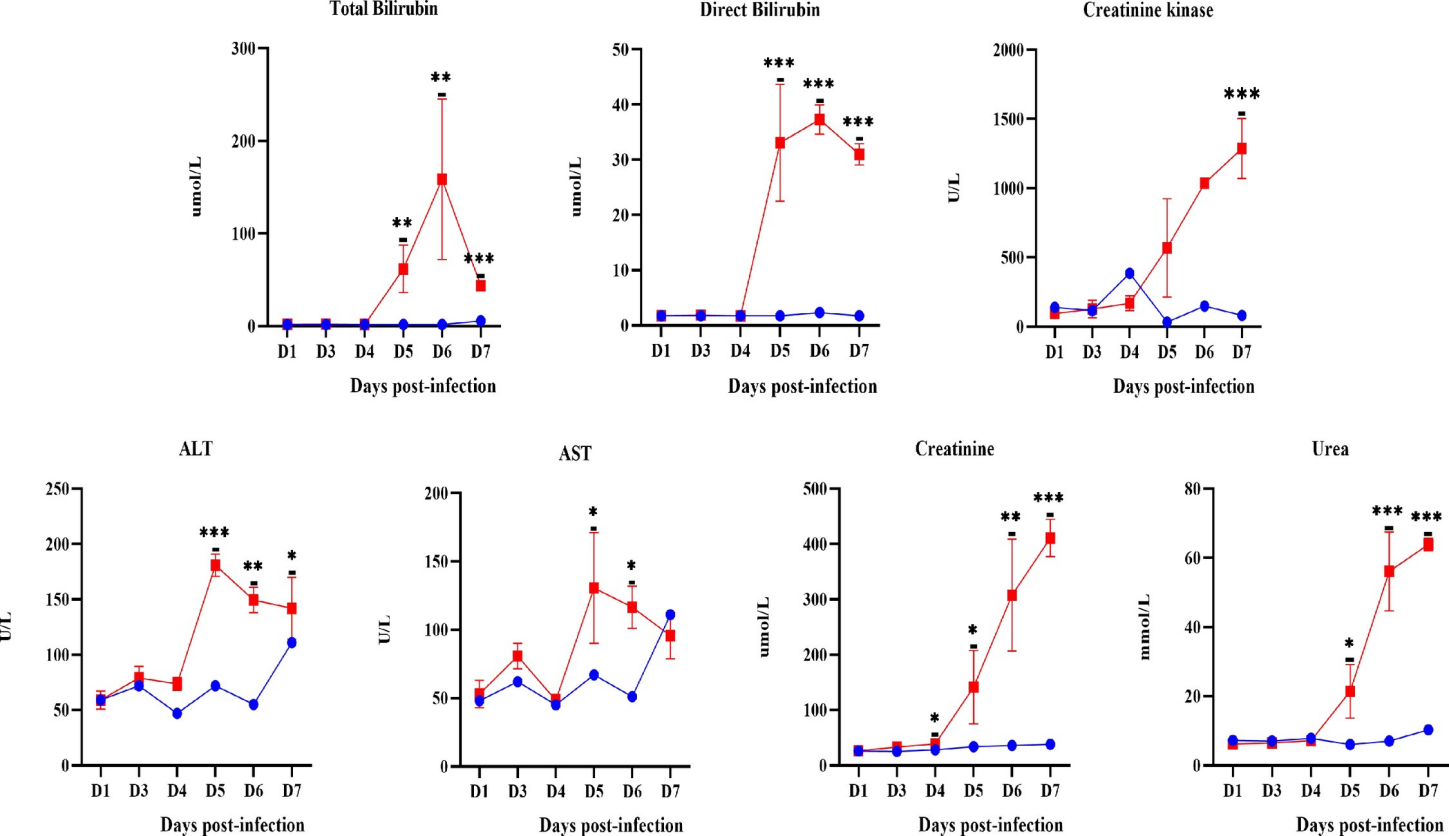
Serum level of total bilirubin, direct bilirubin, CK, ALT, AST, creatinine and urea in control (blue line) and infected hamsters (red lines). The biochemical measurements convey the function state of the liver and kidney. D1-D7: Day 1 to Day 7. *P≤0.05, **P≤0.01, ***P≤0.001.

The level of total bilirubin (TB), direct bilirubin (DB) and creatinine kinase (CK) in infected hamsters showed a significant increase (p-value<0.05) compared to that of control hamsters from day 5 p.i. onwards (Fig 8). Similarly, alanine transaminase (ALT) and aspartate aminotransferase (AST) levels rose significantly (p-value = <0.05) beginning on day 5 p.i. The levels of creatinine and urea in infected hamsters began to increase beginning from day 3 p.i. and 5 p.i. respectively.

### Expression of immune mediators in infected animals

#### Pro-inflammatory mediators

**Fig 9:**
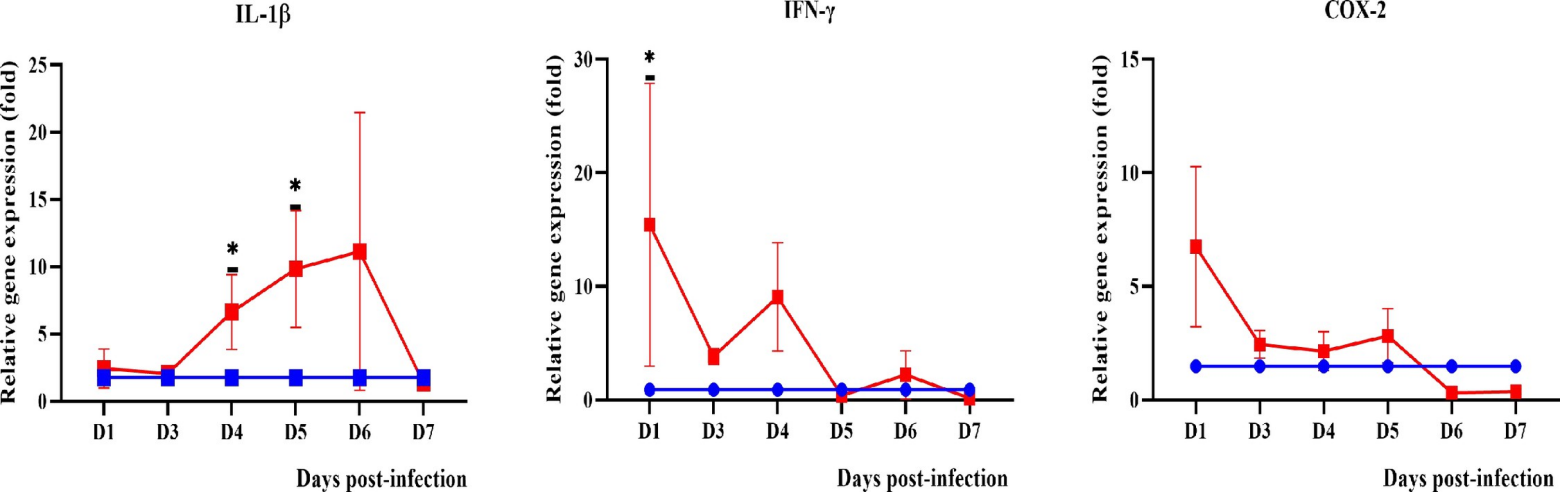
Modulation of pro-inflammatory cytokines and enzyme in the blood of control (blue line) and infected hamsters (red line). Total RNA was extracted from whole blood on day 1 and days 3 to 7 p.i.. D1-D7: Day 1 to day 7. *P≤0.05. The fold gene expression value can be found in S2 Table.

**Fig 10:**
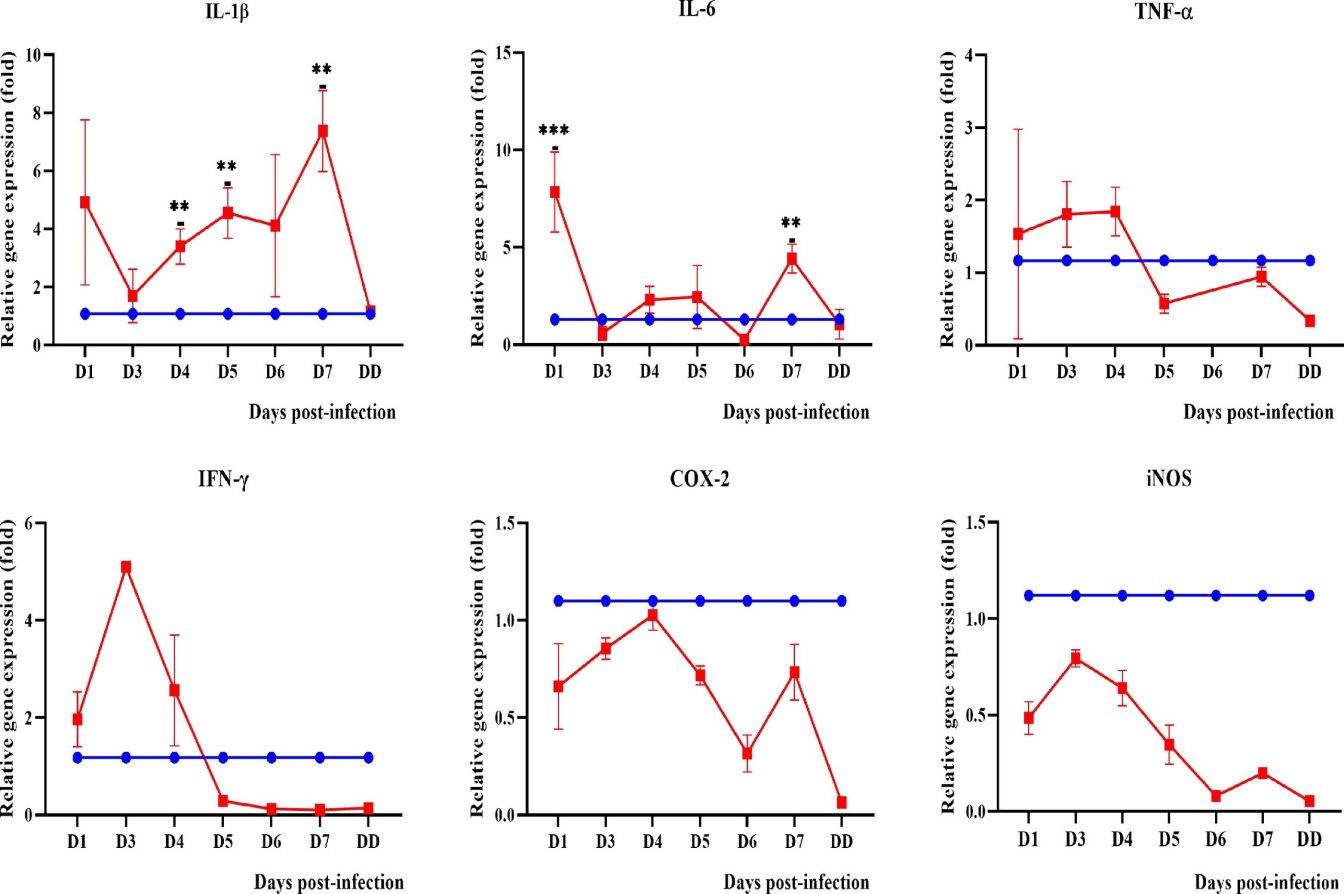
Modulation of pro-inflammatory cytokines and enzymes in the lungs of control (blue line) and infected hamsters (red line). Total RNA was extracted from lungs on day 1 p.i. and days 3 to 7 p.i. D1-D7: Day 1 to day 7. DD: dead. **P≤0.01, ***P≤0.001. The fold gene expression value can be found in S2 Table.

**Fig 11:**
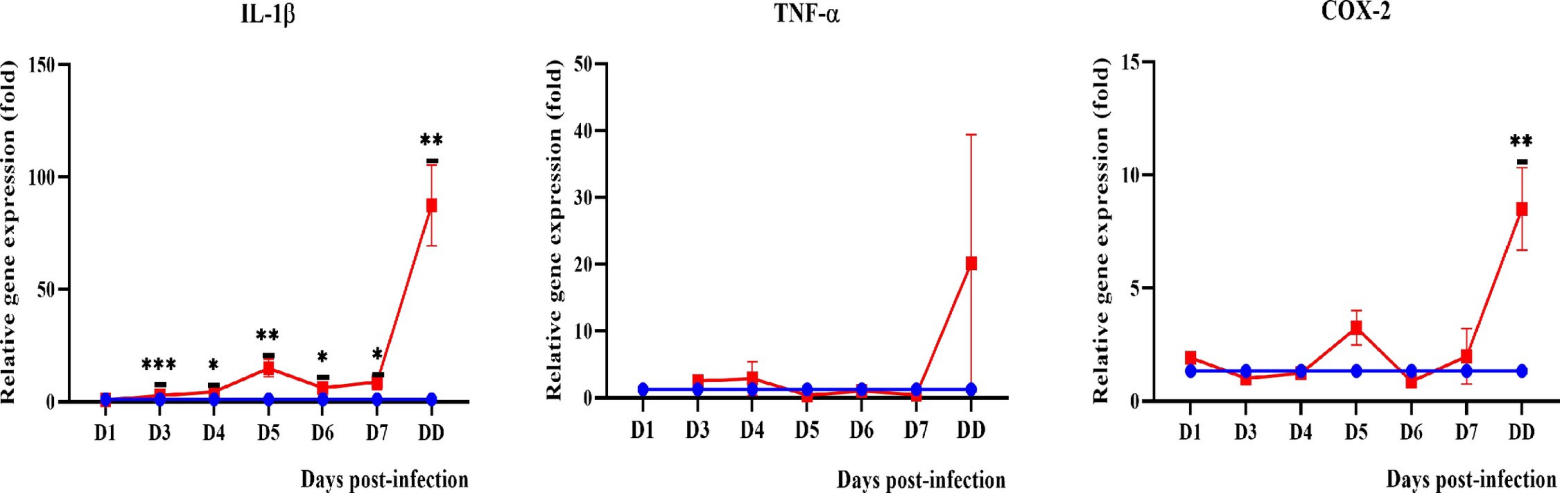
Modulation of pro-inflammatory cytokines and enzymes in the liver of control (blue line) and infected hamsters (red line). Total RNA was extracted from liver on day 1 p.i. and days 3 to 7 p.i. D1-D7: Day 1 to day 7. DD: dead. *P≤0.05, **P≤0.01, ***P≤0.001.The fold gene expression value can be found in S2 Table.

**Fig 12:**
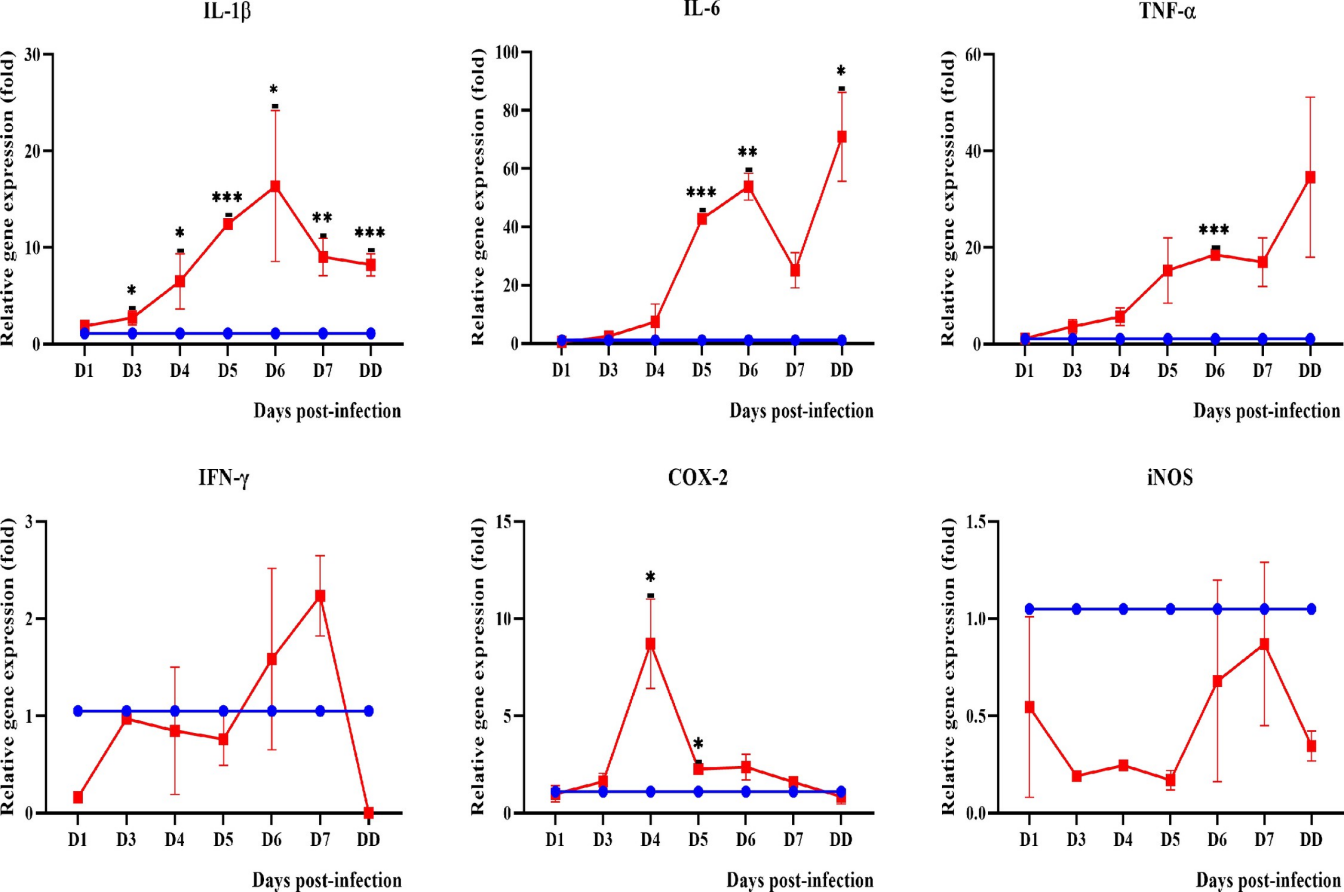
Modulation of pro-inflammatory cytokines and enzymes in the kidneys of control (blue line) and infected hamsters (red line). Total RNA was extracted from kidneys on day 1 p.i. and days 3 to 7 p.i. D1-D7: Day 1 to day 7. DD: dead. *P≤0.05, **P≤0.01, ***P≤0.001. The fold gene expression value can be found in S2 Table.

Among the pro-inflammatory cytokines and enzymes (interleukin-1beta: IL-1β, interleukin-6: IL-6, Tumor necrosis factor alpha: TNF-α, interferon gamma: IFN-γ, cyclooxygenase-2: COX-2 and inducible nitric oxide synthase: iNOS) tested, IL-1β was found to be significantly expressed in blood and all organs (Figs 9–12) from day 3 onwards. Expression of IL-6 and TNF-α was only observed in the lungs and kidneys. IL-6 was significantly higher in the lungs on days 1 and 7 p.i. while in kidneys, it demonstrated a progressive increase from day 3 p.i. TNF-α showed increased expression in the lungs from day 1 to day 4 p.i. while in kidneys, it showed a progressive increase from day 3 p.i. IFN-γ was higher until day 4 p.i. in blood and lungs. COX-2 was higher than the control in blood until day 5 p.i. while in kidneys, it increased progressively from day 3 p.i. In the lungs, COX-2 was found to be downregulated beginning on day 1 p.i. IL-1β, TNF-α and COX-2 were found to be highly expressed in the liver of dead hamsters. iNOS was downregulated in lungs and kidneys and not detected in blood and liver.

#### Chemokines CXCL10/IP-10 and CCL3/MIP-α

**Fig 13:**
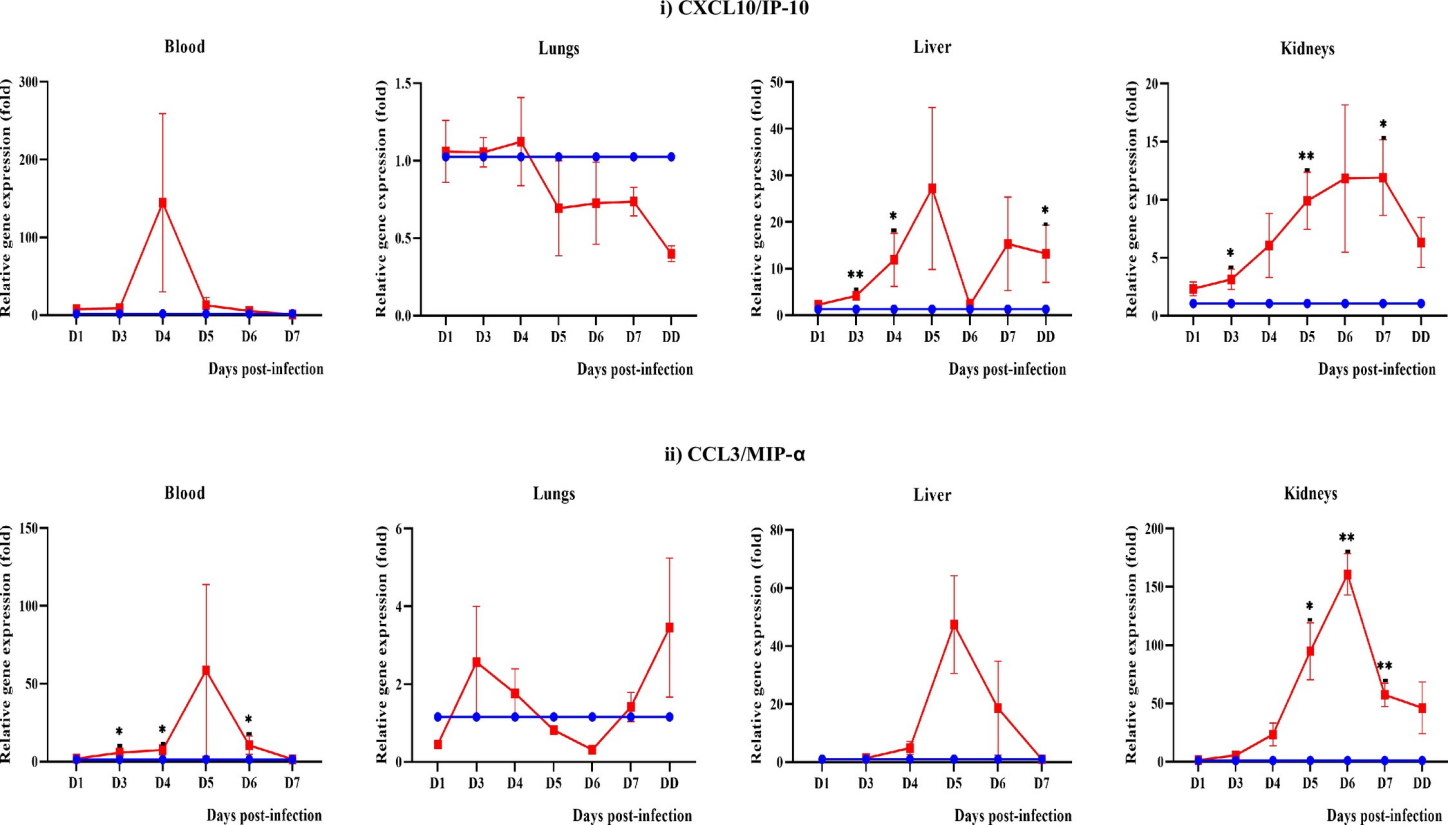
Modulation of chemokines in blood, lungs, liver and kidneys of control (blue line) and infected hamsters (red line). D1-D7: Day 1 to day 7. DD: dead *P≤0.05, **P≤0.01, ***P≤0.001. Control. The fold gene expression value can be found in S3 Table.

Increased expression of C-X-C motif chemokine ligand 10 (CXCL10/IP-10) in the blood (from day 1 to 4 p.i), liver (day 1 to 5 p.i.) and kidneys (day 1 to 7 p.i.) (Fig 13). Likewise, chemokine (C-C motif) ligand 3 (CCL3/MIP-α) expression increased in blood, liver and kidneys.

#### Anti-inflammatory mediators

**Fig 14:**
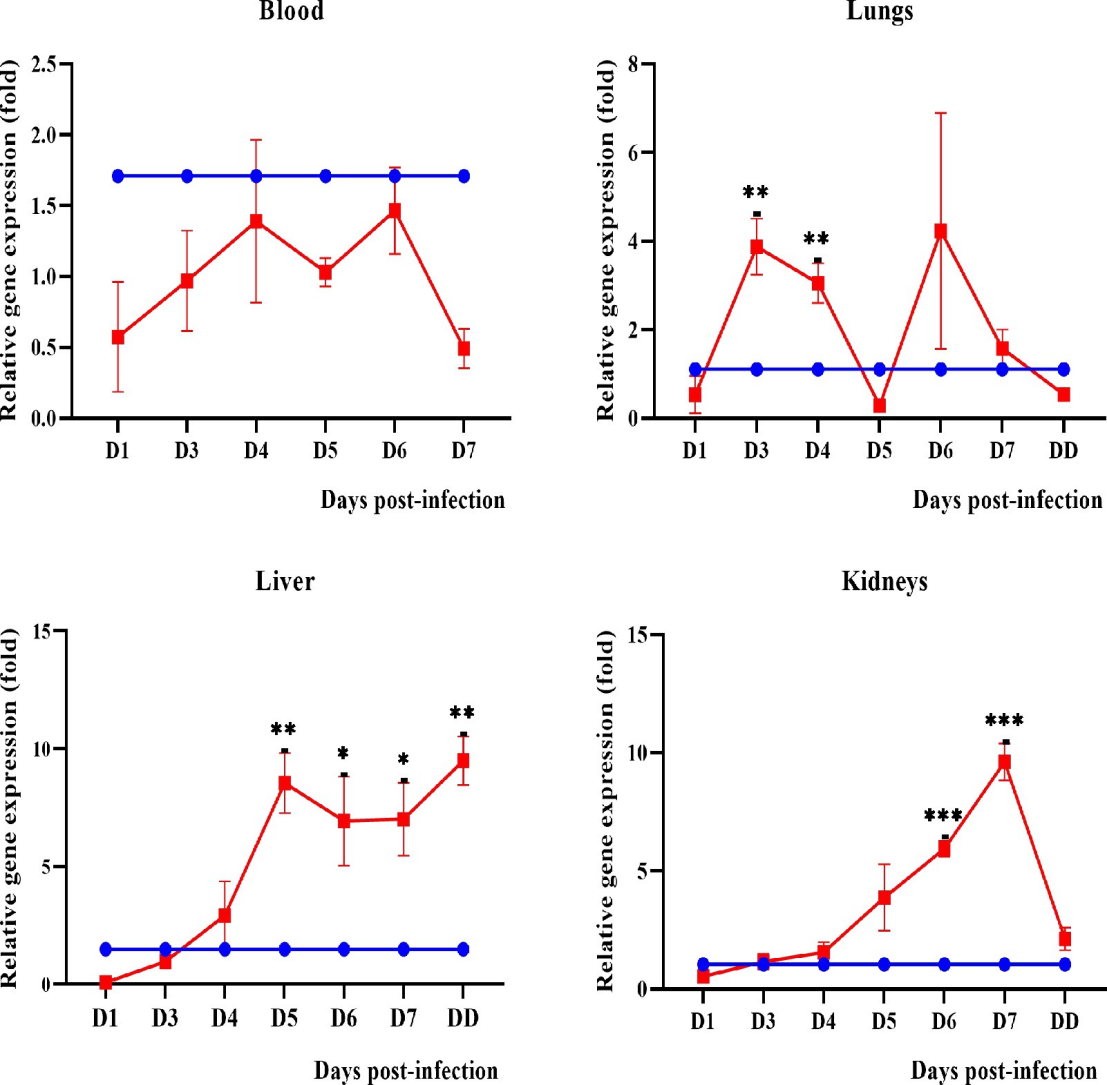
Expression of TGF-β1 in blood, lungs, liver and kidneys of control (blue line) and infected hamsters (red line). D1-D7:Day 1 to day 7. DD: dead. *P≤0.05, **P≤0.01, ***P≤0.001.The fold gene expression value can be found in S4 Table.

The expression of transforming growth factor-beta 1 (TGF-β1) showed an increasing pattern in the liver and kidneys (Fig 14) beginning on day 4 p.i. and significantly high on days 6 and 7 p.i. TGF-β1 was found to be downregulated in blood while in the lungs it was slightly higher on days 3 and 4 p.i. Two hamsters showed amplification of IL-10 in lungs, liver and kidneys with a ct value of > 35 on days 5 and 6 p.i. One dead hamster showed amplification of IL-10 in the lung with a ct value of 33.63.

#### Association between clinical manifestations and pathophysiology in infected hamsters: Identification of biomarkers for severe leptospirosis

As summarized in Table 3, the pathophysiological presentations observed for severe leptospirosis in the hamster model included: (1) occurrence of pulmonary haemorrhage earlier than liver and kidney damages (before any clinical manifestations), (2) increased WBC, monocytes and neutrophils and decreased lymphocytes and platelets (before severe signs and symptoms), (3) serum biochemistry parameters changes were concurrent with the apparent clinical manifestations and (4) earlier expression of pro-inflammatory mediators IL-1β, CXCL10/IP-10 and CCL3/MIP-α in all organs (blood, lungs, liver and kidneys) prior to observable damages. The early expression of IL-1β, CXCL10/IP-10 and CCL3/MIP-α, increase of neutrophils and decrease of lymphocytes and platelets suggesting that these parameters could be used as a cumulative panel for potential biomarkers in severe leptospirosis.

**Table 3:** Summary of disease progression in hamsters infected with *L*. *interrogans* strain HP358.

Parameters	D1	D3	D4	D5	D6	D7
Clinical Signs	.None	.Reduction in body weight	.Reduction in body weight	.Reduction in body weight .Loss of appetite .Reduced physical activity .Developed dyspnea	.Reduction in body weight .Loss of appetite .Reduced physical activity .Dyspnea .3 animals died	.Reduction in body weight .Loss of appetite .Reduced physical activity .Dyspnea .1 animal died
Macroscopy of organs	.Haemorrhage in lungs	.Haemorrhage in lungs	.Haemorrhage in lungs	.Haemorrhage in lungs	.Haemorrhage in lungs .Pale kidney	.Haemorrhage in lungs .Pale kidney
Microscopic (Cumulative score of organ damage)	.Lungs (9) .Liver (3) .Kidney (0	.Lungs (11) .Liver (3) .Kidney (2)	.Lungs (12) .Liver (8) .Kidney (11)	.Lungs (17) .Liver (16) .Kidney (17)	.Lungs (16) .Liver (16) .Kidney (20)	.Lungs (16) .Liver (16) .Kidney (22)
Hemogram	↑WBC ↑Monocytes ↑Neutrophils ↓Lymphocytes ↓ Platelets	↑WBC(*) ↑Monocytes ↑Neutrophils ↓ Lymphocytes ↓Platelets (**)	↑WBC ↑Monocytes ↑Neutrophils ↓ Lymphocytes ↓Platelets	↑WBC(*) ↑Monocytes (***) ↑Neutrophils (***) ↓ Lymphocytes (***) ↓Platelets (***)	↑WBC ↑Monocytes ↑Neutrophils ↓ Lymphocytes ↓Platelets	↑WBC(***) ↑Monocytes(***) ↑Neutrophils(***) ↓ Lymphocytes(***) ↓Platelets
Serum Biochemistry	Same as control	Same as control	↑Creatinine (*)	↑TB (**) ↑DB (***) ↑AST (*) ↑ALT(***) ↑Creatinine (*) ↑Urea (*)	↑TB (**) ↑DB (***) ↑AST (*) ↑ALT(***) ↑Creatinine (*) ↑Urea (*)	↑TB (**) ↑DB (**) ↑CK (***) ↑ALT(*) ↑Creatinine (***) ↑Urea (**)
**Pro-inflammatory and anti-inflammatory markers**						
Blood	↑IL-1β ↑IFN-γ(*) ↑COX-2 ↑CXCL10/IP-1 ↑CCL3/MIP-α	↑IL-1β ↑IFN-γ ↑COX-2 ↑ CXCL10/IP-10 ↑CCL3/MIP-α(*)	↑IL-1β(*) ↑ IFN-γ ↑COX-2 ↑CXCL10/IP-10 ↑CCL3/MIP-α (*)	↑IL-1β(*) ↑COX-2 ↑CXCL10/IP-10, ↑CCL3/MIP-α	↑IL-1β(*) ↑IFN-γ ↑CXCL10/IP-10, ↑CCL3/MIP-α (*)	↑CCL3/MIP-α
Lungs	↑IL-1β ↑IL-6(**)↑↑TNF-α ↑IFN-γ ↑CXCL10/IP-10	↑IL-1β ↑TNF-α ↑IFN-γ ↑CCL3/MIP-α ↑TGF-β1(**)	↑IL-1β(**)↑↑IL-6 ↑TNF-α ↑IFN-γ ↑CXCL10/IP-10 ↑CCL3/MIP-α ↑TGF-β1(**)	↑ IL-1β(**) ↑IL-6	↑IL-1β ↑TGF-β1	↑CCL3/MIP-α ↑TGF-β1
Liver	↑COX-2 ↑CXCL10/IP-10	↑IL-1β(***) ↑ TNF-α ↑CXCL10/IP-10(**) ↑CCL3/MIP-α ↑TGF-β1	↑IL-1β(*) ↑TNF-α ↑CXCL10/IP-10(*) ↑CCL3/MIP-α ↑TGF-β1	↑IL-1β(**) ↑COX-2 ↑CXCL10/IP-10 ↑CCL3/MIP-α ↑TGF-β1(**)	↑ IL-1β(*) ↑CCL3/MIP-α ↑TGF-β1(*)	↑IL-1β(**)↑COX-2 ↑CXCL10/IP-10 ↑TGF-β1 (*)
Kidneys	↑IL-1β ↑TNF-α ↑CXCL10/IP-10 ↑CCL3/MIP-α	↑IL-1β(*) ↑IL-6 ↑TNF-α ↑ COX-2 ↑CXCL10/IP-10(*) ↑CCL3/MIP-α ↑TGF-β1	↑IL-1β(*) ↑IL-6 ↑TNF-α ↑COX-2(*) ↑CXCL10/IP-10 ↑CCL3/MIP-α ↑TGF-β1	↑IL-1β(***) ↑IL-6(***) ↑TNF-α, COX-2(*) ↑CXCL10/IP-10(**)↑CCL3/MIP-α(*) ↑TGF-β1	↑IL-1β(*) ↑IL-6(**) ↑TNF-α(***) ↑COX-2 ↑CXCL10/IP-10 ↑CCL3/MIP-α(**) ↑TGF-β1(***)	↑IL-1β(**) ↑IL-6, TNF-α ↑COX-2 ↑CXCL10/IP-10(*) ↑CCL3/MIP-α(**) ↑TGF-β1(***)

Note

-The number of indicated in microscopic organ observation is the cumulative score of organs damage (Table 2)

-↑: Increase

-Only immune mediators showing expression are included.

(*):P≤0.05

(**):P≤0.01

(***):P≤0.001

-WBC: White blood cell

-ALT: Alanine transaminase

-AST: Aspartate aminotransferase

-TB: Total bilirubin

-DB: Direct bilirubin

-IL-1β: interleukin-1β

-TNF-α: Tumor necrosis factor alpha

-IL-6: interleukin-6

-COX-2: cyclooxygenase-2

-CXCL10/IP-10: C-X-C motif chemokine ligand 10/ interferon gamma-induced protein 10

-CCL3/MIP-α: Chemokine (C-C motif) ligand 3 (CCL3)/macrophage inflammatory protein 1-alpha

-TGF-β1: Transforming growth factor beta

## Discussion

Leptospirosis presents a protean clinical manifestation and most cases (90%) are mild. Severe cases account for 5 to 15% and usually occur in the immune phase of illness [12,24,25]. Severe leptospirosis also presents with a fulminant monophasic illness [26–28]. In both conditions, the evolution of the disease is rapid and potentially fatal if not treated. Hence, early management of the disease is vital. Given the fact that the prompt diagnosis of this illness is challenging and the sudden progression to severe leptospirosis is life-threatening; understanding the sequence of disease progression and determination of early prognosis markers are of utmost importance for a favourable outcome.

In the present study, the first investigation was focused on the clinical manifestation developed in the hamsters when infected with the *L*. *interrogans* strain HP358. Hamsters reproduce the severe form of human leptospirosis [29] thereby suitable to be used as a model for studying the progression of severe leptospirosis. As seen from the results, loss of body weight started from day 3 p.i. and from day 5 p.i., all hamsters showed loss of appetite, reduced physical activities and difficulty in breathing (dyspnea). Similar clinical signs were also reported in several previous studies [19,30,31].

To relate the above clinical manifestations with the sequence of events occurring within the body during the infection, four hamsters (one control and three infected) were euthanized on days 1 (24 hours post-infection), 3, 4, 5, 6 and 7 p.i. The earliest pathological changes (macroscopically and microscopically) observed in the infected hamsters were pulmonary haemorrhage and blood vessel congestion in the lungs, liver and kidneys. The earliest sign of haemorrhage was observed in the lungs from day 1 p.i. while in kidneys on day 4 p.i. Marked organ damages (Table 3) were detected beginning from day 4 p.i concurrent with the clinical manifestations. These were followed by the death of four hamsters on days 6 and 7 p.i. while progressive moribund conditions were observed in the remaining hamsters. A similar observation was reported in a recent study where pulmonary haemorrhage appeared much earlier followed by liver and renal damages prior to the animal’s state of moribund and death [31]. In human leptospirosis, pulmonary haemorrhage is the severe form of the illness, though it occurs only in a small number of cases, mortality is seen higher among these patients (more than 70%) [32,33]. The ability of the leptospires to invade multiple organs also depends on the *Leptospira* species or strain [16]. It could be postulated that in patients with severe leptospirosis, the patients might be infected with a highly virulent *Leptospira* strain invading multiple organs. As observed in the present study, the lungs were the first organ showing damage, hence it is important to monitor the respiratory problems or bleeding in leptospirosis patients as a prognostic factor for severe leptospirosis. As reported in previous studies, pulmonary haemorrhage could occur prior to jaundice and renal failure and led to severe disease and fatality in human leptospirosis [34,35].

Leptospiral DNA was detected in the blood and all organs on day 1 p.i. indicating rapid dissemination and successful colonization of *L*. *interrogans* strain HP358 in the hamster as reported in other studies [17,36]. The bacterial load in blood and all organs continued to increase until day 5 p.i. denoting the replication of leptospires. We observed a decrease in the leptospiral load from day 6 p.i. onwards in blood, lungs and liver while it was maintained in kidneys. Hamsters that died before the completion of the study had a high load of leptospires in all organs (lungs, liver and kidneys) compared to those euthanized on day 7 p.i.

Changes in haematological parameters were observed to occur as early as on day 1 p.i. indicating the response of the innate immunity of hamsters against the invading leptospires. Monocytes and neutrophils continued to increase while lymphocytes and platelets showed decreasing trends. Although neutrophils and monocytes could recognize leptospires, both have limited capacity to control the pathogen [37–42]. It was reported that pathogenic *Leptospira* spp. could bind to platelets and induce cytotoxic effects resulting in dysfunction and clearance of platelets [43–47]. A low level of platelets noticed in the present study could also play important role in the haemorrhagic presentation as observed in both animal [46] and human leptospirosis [48–50]. Significant neutrophilia and lymphocytopenia had also been reported in severe and fatal cases of human leptospirosis [6,51–55]. The significant changes in total and direct bilirubin, AST, ALT, creatinine, urea and CK appeared much later than the haematological parameters which were on days 4 and 5 p.i. henceforth concurrent with the appearance of damage in the liver and kidneys. These changes in the liver and kidney function tests were in agreement with changes in human leptospirosis [51,55–59].

Both pro-inflammatory cytokines and chemokines were expressed in the infected hamsters. Pro-inflammatory cytokines and enzymes which were IL-1β, IL-6, TNF-ɑ, IFN-γ and COX-2 and chemokines CXCL10/IP-10 and CCL3/MIP-α showed upregulation as reported in both human and animal leptospirosis [6,17,18,23,60–62]. Pro-inflammatory enzyme iNOS was downregulated in lungs and kidneys and not expressed in blood and liver which was similarly reported in a previous study [63]. Anti-inflammatory cytokine TGF-β1 showed expression from day 3 p.i. while IL-10 was slightly induced in some of the dead hamsters on days 5 and 6 p.i. as similarly reported in the previous study [64].

Overall, two main manifestations of pathophysiology in severe leptospirosis were observed; haemorrhage and organ damage where pulmonary haemorrhage appeared as the earliest pathological event. The mechanism of pulmonary haemorrhage is still poorly understood and could result from multiple factors [65]. Direct injury by leptospires or their circulating products (leptospiral outer membrane proteins, glycoproteins, hemolysins and lipopolysaccharides) and indirectly by the host’s immune dysregulation have been proposed to contribute to the haemorrhagic manifestation in leptospirosis [66–69]. Pathogenic leptospires could bind to the endothelial lining of the blood vessels [50,67,70–72] and potentially disrupts the endothelial cell layer [69,73]. IL-6 has been associated with severe pulmonary haemorrhage [6]. In the present study, IL-6 was significantly high in the lung on day 1 p.i. and while in the kidney, it only appears on day 4 p.i. concurrent with the haemorrhagic presentation, thereby could support the role of this cytokine in the haemorrhagic presentation in leptospirosis.

Haemorrhagic manifestation of leptospirosis could also be due to vascular cell damage by reactive oxygen species (ROS) and arterial hypertension [74]. Neutrophils could produce ROS [75], thus the high production of neutrophils in the infected animals may indirectly contribute to the haemorrhagic manifestation observed in this study. Nitric oxide (NO) production catalyzed by the enzyme iNOS [76,77] functions as a vasodilator [78] and is also able to control the production and activity of ROS [79,80] in inhibiting the replication of the pathogen [81–83]. The down-regulation of iNOS influences the release and activities of NO [76,77]. Low or specific inhibition of iNOS is associated with pulmonary haemorrhage [63], increased mortality, bacterial load in the kidney and reduced specific humoral response [84] in the hamster model and patients with severe disease [85].

Mild alveoli dilation observed on day 5 p.i. is a contributing factor for dyspnea which is in agreement with a report in a recent study [86]. The dilated alveolus is a characteristic of chronic obstructive pulmonary disease (COPD) with dyspnea as the cardinal symptom. Enlargement of the alveolus destructs the alveoli walls through inflammation [87]. Cytokine IL-1β has been shown to exert airway inflammation and emphysema in the COPD mice model [88–92]. The raised level of serum IL-1β in patients with mild alveolar dilation is in agreement with the present investigation where expression of IL-1β was significantly high from day 4 p.i. onwards [93].

The progression of damage in the liver and kidneys is associated with the changes in the serum biochemistry and increased expression of inflammatory cytokines and chemokines. The liver and kidney functions test markers (total bilirubin, direct bilirubin, ALT, AST, creatinine and urea, CK) showed a significant increase from day 4 p.i. The progressive upregulation of inflammatory cytokines and chemokines in the kidney (IL-1β, IL-6, TNF-ɑ, COX-2, TGF-β1, CXCL10/IP-10 and CCL3/MIP-α) and liver (IL-1β, TGF-β1, CXCL10/IP-10 and CCL3/MIP-α) beginning from day 3 p.i. support the possibility that damage in these organs is associated with the increased inflammatory response. CXCL10/IP-10 and CCL3/MIP-α are known to mediate the migration of T cells, monocytes, neutrophils and natural killer (NK) cells from the bloodstream to tissues in response to inflammation [94–97].

A severe manifestation of leptospirosis is comparable to sepsis that occurs due to an imbalance in the inflammatory responses in the host infected with pathogens. The infected hosts release inflammatory mediators in an attempt to neutralize the pathogenic effect. The occurrence of a sustained and increased expression of pro-inflammatory cytokines characteristic of a “cytokine storm” will lead to persistent inflammation [9] and this is followed by a massive and systemic production of anti-inflammatory mediators resulting in a state of “immunoparalysis” and tissue oedema [98,99]. Tissues oedema could impair the local organ perfusion leading to loss of organ function and endothelial permeabilization [98,100]. In asymptomatic or mild leptospirosis and mice animal models, homeostasis between pro-inflammatory and anti-inflammatory is maintained where both are produced early and strictly regulated [9,17]. In severe leptospirosis in humans, two scenarios have been reported; either high IL-10 and low TNF-α [6,61] or low IL-10 and high TNF-α [17,101,102]. In this present study, we saw a sudden surge of the pro-inflammatory mediators (cytokines and chemokines) beginning from day 3 p.i.without the prominent expression of anti-inflammatory IL-10. A low expression of IL-10 (ct value of >33 cycles) and early (day 3) expression of TNF-α, TGF-β and IP-10 in hamsters infected with *L*. *interrogans* serovar *Pyrogenes* has been reported earlier [64]. In conclusion, severe leptospirosis due to the *L*. *interrogans* strain HP358 could be characterized as a sudden and increased pro-inflammatory response with delayed and significantly low expression of anti-inflammatory IL-10. The severe leptospirosis characterized in the hamster model in the present study is in accordance with the severe form of leptospirosis in humans where patients showed mild symptoms during the early course of the disease and developed a rapidly worsening condition leading to fatality within 72 hours [70,103]. As observed in this study, the rapid evolution to severe illness and fatality in hamsters occurred when most inflammatory mediators were expressed and all organs (lungs, liver and kidneys) were affected. Likewise, the severe form of human leptospirosis involves haemorrhage and multiple organ damages.

Identification of biomarkers in leptospirosis is important not only for diagnosis but also to predict the progression to severity. The main characteristic of ideal biomarkers is their early detection [104] for timely intervention in patients management. A panel of biomarkers will increase the specificity and sensitivity of the diagnosis compared to a single biomarker [101,105]. Serum biochemistry may not be a good predictor for severe leptospirosis as these markers are detected only after the occurrence of serious damage to the liver and kidneys. Cytokines play a major role in host-pathogen interaction and prognosis [18]. Several earlier studies have identified significant expression of IL-1β, IL-2, IL-4, IL-6, IL-8, IL-10 and TNF-α in severe leptospirosis [6,102,106]. However, these studies were conducted in one-time sampling. Progressive monitoring is important to elucidate the progression of cytokines levels and to determine the most appropriate biomarker for disease severity. As the damage in leptospirosis surge rapidly, we recommend performing blood and cytokines profiling at 24 hours interval to monitor the biomarkers for the severe illness that could prevent substantial damage to the organs. From the present investigation, we found the expression of IL-1β, CXCL10/IP-10 and CCL3/MIP-α increased in the blood and most organs day by day as the infection progressed. On the other hand, neutrophils increased progressively from day 1 p.i. while lymphocytes and platelets showed a declining trend. Taken all these data together, we suggest that high levels of IL-1β, CXCL10/IP-10, CCL3/MIP-α, neutrophils and low levels of lymphocytes and platelets could serve as a cumulative panel of potential biomarkers in the disease progression from mild to severe in leptospirosis. As this study was conducted in an animal model, a progressive validation study in human leptospirosis is recommended.

## Conclusion

Severe leptospirosis is characterized by a sudden over-expression of pro-inflammatory cytokines after infection of *L*. *interrogans* strain HP358 and without prominent expression of regulatory cytokines. The massive expression of cytokines and chemokines led to sudden and rapid damage to the liver and kidneys. Damages in the lungs, liver and kidneys were preceded by the early occurrence of haemorrhage in the lungs. High levels of IL-1β, CXCL10/IP-10, CCL3/MIP-α, neutrophils and low levels of lymphocytes and platelets might serve as a cumulative panel of biomarkers in severe leptospirosis.

## Supporting information

S1 Table Leptospiral copies number in blood and organs. (DOC)

S2 Table Fold gene expression value of pro-inflammatory cytokines. (DOC)

S3 Table Fold gene expression value of pro-inflammatory chemokines. (DOC)

S4 Table Fold gene expression value of anti-inflammatory cytokines. (DOC)
