# Supplementary material for: Pulmonary haemorrhage as the earliest sign of severe leptospirosis in hamster model challenged with Leptospira interrogans strain HP358
Source: PLoS Negl Trop Dis. 2022 May 18;16(5):e0010409. doi: 10.1371/journal.pntd.0010409 (PMC9116642; doi:10.1371/journal.pntd.0010409)
Supplement: S1 Table — (DOC) [file pntd.0010409.s001.doc]

**S1 Table: Leptospiral Copies Number in Blood and Organs**

| **Day** | **ID Sample** | **Blood** | **Lungs** | **Liver** | **Kidneys** |
| --- | --- | --- | --- | --- | --- |
| 1 | C1 | - | - | - | - |
|  | I2 | NA | 9.34 x 10^2^ | 2.61 x 10^5^ | 2.52 x 10^3^ |
|  | I3 | 5.74 x 10^4^ | 1.39 x 10^3^ | 2.05 x 10^5^ | 1.52 x 10^4^ |
|  | I4 | 2.28 x 10^3^ | 6.55 x 10^2^ | 1.46 x 10^5^ | 1.63 x 10^3^ |
|  | **Average** | **2.98 x 10^4^** | **9.93 x 10^2^** | **2.04 x 10^5^** | **6.45 x 10^3^** |
| 3 | C5 | - | - | - | - |
|  | I6 | 2.80 x 10^5^ | 1.09 x 10^4^ | 1.36 x 10^6^ | 1.08 x 10^5^ |
|  | I7 | 5.34 x 10^4^ | 1.55 x 10^5^ | 7.44 x 10^6^ | 8.71 x 10^5^ |
|  | I8 | 1.34 x 10^5^ | 8.78 x 10^3^ | 1.32 x 10^6^ | 3.02 x 10^5^ |
|  | **Average** | **1.56 x 10^5^** | **5.82 x 10^4^** | **3.37 x 10^6^** | **4.27 x 10^5^** |
| 4 | C9 | - | - | - | - |
|  | I10 | 2.02 x 10^4^ | 2.93 x 10^5^ | 4.91 x 10^6^ | 1.12 x 10^7^ |
|  | I11 | 1.73 x 10^5^ | 7.39 x 10^4^ | 4.20 x 10^6^ | 1.56 x 10^6^ |
|  | I12 | 1.60 x 10^6^ | 1.48 x 10^5^ | 6.66 x 10^6^ | 2.53 x 10^6^ |
|  | **Average** | **5.97 x 10^5^** | **1.72 x 10^5^** | **5.26 x 10^6^** | **5.1 x 10^6^** |
| 5 | C13 | - | - | - | - |
|  | I14 | 2.01 x 10^7^ | 1.90 x 10^7^ | 1.25 x 10^8^ | 6.22 x 10^7^ |
|  | I15 | 3.10 x 10^4^ | 1.51 x 10^6^ | 5.57 x 10^7^ | 7.44 x 10^7^ |
|  | I16 | 5.39 x 10^4^ | 2.98 x 10^5^ | 1.66 x 10^7^ | 3.76 x 10^7^ |
|  | **Average** | **6.73 x 10^6^** | **6.9 x 10^6^** | **6.58 x 10^7^** | **5.8 x 10^7^** |
| 6 | C17 | - | - | - | - |
|  | I18D | NA | 6.87 x 10^6^ | 2.87 x 10^8^ | 3.32 x 10^7^ |
|  | I19D | NA | 5.51 x 10^6^ | 1.34 x 10^8^ | 7.01 x 10^7^ |
|  | I20D | NA | 7.55 x 10^6^ | 5.04 x 10^7^ | 3.22 x 10^7^ |
|  | I21 | 1.42 x 10^4^ | 2.19 x 10^3^ | 1.08 x 10^5^ | 5.07 x 10^7^ |
|  | I22 | NA | 5.38 x 10^4^ | 1.86 x 10^7^ | 6.01 x 10^7^ |
|  | **Average** | **1.42 x 10^4^** | **3.99 x 10^6^** | **9.80 x 10^7^** | **4.93 x 10^7^** |
| 7 | C23 | - | - | - | - |
|  | I24D | NA | 4.94 x 10^3^ | 3.57 x 10^5^ | 2.73 x 10^7^ |
|  | I25 | 3.43 x 10^2^ | 9.80 x 10^1^ | 1.54 x 10^4^ | 9.09 x 10^6^ |
|  | I26 | 5.14 x 10^2^ | 1.47 x 10^3^ | 4.22 x 10^5^ | 2.07 x 10^7^ |
|  | I27 | 3.14 x 10^3^ | 3.09 x 10^2^ | 8.66 x 10^3^ | 3.41 x 10^6^ |
|  | **Average** | **1.33 x 10^3^** | **1.70 x 10^3^** | **2.01 x 10^5^** | **1.5 x 10^7^** |

*C1, C5, C9, C13, C17, C23 = Control hamsters

*I = Infected hamsters

*I18D, I19D, I20D, I24D = Infected hamsters that died during the study period

*NA= Sample was not available
