# Supplementary material for: Pulmonary haemorrhage as the earliest sign of severe leptospirosis in hamster model challenged with Leptospira interrogans strain HP358
Source: PLoS Negl Trop Dis. 2022 May 18;16(5):e0010409. doi: 10.1371/journal.pntd.0010409 (PMC9116642; doi:10.1371/journal.pntd.0010409)
Supplement: S2 Table — (DOC) [file pntd.0010409.s002.doc]

| **Gene**  **S2 Table: Fold gene expression value of pro-inflammatory cytokines** | **Time (Days)** | **Blood** | **Lungs** | **Liver** | **Kidneys** |
| --- | --- | --- | --- | --- | --- |
| IL-1β | Control | 1.752 ± 1.44 | 1.08 ± 0.49 | 1.06 ± 0.41 | 1.07 ± 0.43 |
|  | 1 | 2.43 ± 1.28 | 3.74 ± 1.50 | 0.83 ± 0.49 | 1.89 ± 0.41 |
|  | 3 | 2.07 ± 0.87 | 1.69 ± 0.61 | 2.84 ± 0.25 (***) | 2.71 ± 0.58 (*) |
|  | 4 | 6.63 ± 2.01 (*) | 3.40 ± 0.53 (**) | 4.39 ± 0.96 (*) | 6.50 ± 2.10 (*) |
|  | 5 | 9.83 ± 2.97 (*) | 4.55 ± 0.70 (**) | 14.99 ± 3.29 (**) | 12.43 ± 0.47 (***) |
|  | 6 | 11.14 ± 4.98 | 4.12 ± 1.35 | 6.16 ± 1.34 (*) | 16.37 ± 4.15 (*) |
|  | 7 | 1.35 ± 0.93 | 7.37 ± 1.06 (**) | 8.87 ± 2.85 (*) | 9.03 ± 1.44 (**) |
|  | DD | NA | 1.08 ± 0.32 | 87.38 ± 15.06 (**) | 8.21 ± 1.02 (***) |
| IL-6 | Control | 1.1 ± 0.65 | 1.29 ± 0.66 | - | 1.13 ± 0.75 |
|  | 1 | 0.23 ± 0.00 | 7.85 ± 1.09 (***) | - | 0.52 ± 0.55 |
|  | 3 | - | 0.59 ± 0.52 | - | 2.48 ± 1.45 |
|  | 4 | 2.57 ± 2.32 | 2.30 ± 0.60 | - | 7.45 ± 7.89 |
|  | 5 | 0.04 ± 0.00 | 2.45 ± 1.13 | - | 42.75 ± 1.74 (***) |
|  | 6 | 0.6 ± 0.78 | 0.24 ± 0.49 | - | 53.84 ± 4.69 (**) |
|  | 7 | 0.03 ± 0.00 | 4.42 ± 0.62 (**) | - | 25.16 ± 7.81 |
|  | DD | NA | 1.04 ± 0.64 | - | 70.92 ± 19.61 (*) |
| TNF-α | Control | 1.14 ± 0.61 | 1.17 ± 0.69 | 1.28 ± 1.12 | 1.07 ± 0.54 |
|  | 1 | - | 1.54 ± 1.03 | - | 1.17 ± 0.63 |
|  | 3 | - | 1.81 ± 0.59 | 2.52 ± 0.69 | 3.61 ± 1.93 |
|  | 4 | - | 1.84 ± 0.49 | 2.88 ± 3.33 | 5.68 ± 2.36 |
|  | 5 | 0.76 ± 0.79 | 0.57 ± 0.43 | 0.39 ± 0.64 | 15.26 ± 8.73 |
|  | 6 | 0.65 ± 0.17 | - | 1.00 ± 0.95 | 18.51 ± 0.39 (***) |
|  | 7 | 0.63 ± 0.43 | 0.94 ± 0.43 | 0.46 ± 0.00 | 16.99 ± 6.48 |
|  | DD | NA | 0.34 ± 0.52 | 85.18 ± 85.2 | 34.59 ± 24.86 |
| IFN-γ | Control | 0.89 ± 0.86 | 1.18 ± 0.70 | - | 1.06 ± 0.52 |
|  | 1 | 15.43 ± 10.44 (*) | 1.97 ± 0.67 | - | 0.16 ± 0.37 |
|  | 3 | 3.84 ± 0.77 | 5.1 ± 0.00 | - | 0.97 ± 0.00 |
|  | 4 | 9.08 ± 2.98 | 2.56 ± 1.21 | - | 0.85 ± 0.75 |
|  | 5 | 0.39 ± 0.66 | 0.29 ± 0.53 | - | 0.67 ± 0.57 |
|  | 6 | 2.21 ± 1.46 | 0.12 ± 0.00 | - | 0.76 ± 0.44 |
|  | 7 | NA | 0.10 ± 0.53 | - | 2.24 ± 0.59 |
|  | DD | NA | 0.14± 0.53 | - | - |
| COX-2 | Control | 1.49 ± 1.19 | 1.11 ± 0.25 | 1.34 ± 1.03 | 1.07 ± 0.43 |
|  | 1 | 6.74 ± 2.41 | 0.66 ± 0.23 | 1.92 ± 0.65 | 0.98 ± 0.43 |
|  | 3 | 2.45 ± 0.82 | 0.86 ± 0.19 | 1.00 ± 0.65 | 1.62 ± 0.44 |
|  | 4 | 2.15 ± 0.91 | 1.03 ± 0.16 | 1.23 ± 0.78 | 8.72 ± 1.94 (*) |
|  | 5 | 2.82 ± 1.06 | 0.72 ± 0.16 | 3.24 ± 0.83 | 2.27 ± 0.35 (*) |
|  | 6 | 0.32 ± 0.89 | 0.32 ± 0.19 | 0.87 ± 0.78 | 2.37 ± 0.52 |
|  | 7 | 0.37 ± 0.72 | 0.73 ± 0.19 | 1.98 ± 1.01 | 1.59 ± 0.33 |
|  | DD | NA | 0.06 ± 0.15 | 8.50 ± 1.47 (**) | 0.84 ± 0.43 |
| INOS | Control | - | 1.12 ± 0.63 | - | 1.04 ± 0.39 |
|  | 1 | - | 0.49 ± 0.48 | - | 0.55 ± 0.54 |
|  | 3 | - | 0.79 ± 0.47 | - | 0.19 ± 0.00 |
|  | 4 | - | 0.64 ± 0.38 | - | 0.25 ± 0.27 |
|  | 5 | - | 0.35 ± 0.39 | - | 0.17 ± 0.27 |
|  | 6 | - | 0.08 ± 0.47 | - | 0.68 ± 0.59 |
|  | 7 | - | 0.20 ± 0.38 | - | 0.87 ± 0.58 |
|  | DD | - | 0.05 ± 0.38 | - | 0.35 ± 0.20 |

**Note:**

DD= Died hamsters

NA=Sample was not available

(*) = P≤0.05

(**) = P≤0.01

(***) =P≤0.001
