# Supplementary material for: Pulmonary haemorrhage as the earliest sign of severe leptospirosis in hamster model challenged with Leptospira interrogans strain HP358
Source: PLoS Negl Trop Dis. 2022 May 18;16(5):e0010409. doi: 10.1371/journal.pntd.0010409 (PMC9116642; doi:10.1371/journal.pntd.0010409)
Supplement: S3 Table — (DOC) [file pntd.0010409.s003.doc]

**S3 Table: Fold gene expression value of pro-inflammatory chemokines**

| **Gene** | **Time (Days)** | **Blood** | **Lungs** | **Liver** | **Kidneys** |
| --- | --- | --- | --- | --- | --- |
| CXCL10/IP-10 | Control | 1.60 ± 1.51 | 1.02 ± 0.24 | 1.32 ± 0.91 | 1.05 ± 0.39 |
|  | 1 | 7.66 ± 3.00 | 1.06 ± 0.21 | 2.27 ± 0.75 | 2.32 ± 0.56 |
|  | 3 | 8.53 ± 3.62 | 1.06 ± 0.19 | 4.19 ± 0.65 (**) | 3.13 ± 0.77 (*) |
|  | 4 | 144.5 ± 75.05 | 1.12 ± 0.25 | 11.90 ± 4.20 (*) | 6.06 ± 2.31 |
|  | 5 | 12.66 ± 6.61 | 0.69 ± 0.27 | 27.21 ± 12.69 | 9.91 ± 2.07 (**) |
|  | 6 | 5.34 ± 2.47 | 0.73 ± 0.23 | 2.22 ± 0.91 | 11.84 ± 3.90 |
|  | 7 | 0.65 ± 0.92 | 0.74 ± 0.16 | 15.32 ± 7.36 | 11.91 ± 2.74 (*) |
|  | DD | NA | 0.40 ± 0.15 | 13.21 ± 4.53 (*) | 6.32 ± 2.17 |
| CCL3/MIP-α | Control | 1.25 ± 0.85 | 1.16 ± 0.77 | 1.04 ± 0.36 | 1.17 ± 0.67 |
|  | 1 | 1.83 ± 1.19 | 0.45 ± 0.58 | - | 1.41 ± 0.61 |
|  | 3 | 5.72 ± 1.44 (*) | 2.57 ± 0.95 | 1.38 ± 0.43 | 5.59 ± 2.01 |
|  | 4 | 7.41 ± 2.43 (*) | 1.77 ± 0.65 | 4.88 ± 2.26 | 23.47 ± 9.73 |
|  | 5 | 58.57 ± 29.22 | 0.82 ± 0.47 | 47.44 ± 16.79 | 94.84 ± 24.38 (*) |
|  | 6 | 10.44 ± 3.17 (*) | 0.31 ± 0.58 | 18.61 ± 12.06 | 160.80 ± 13.25 (**) |
|  | 7 | 1.49 ± 0.69 | 1.41 ± 0.54 | - | 57.35 ± 10.08 (**) |
|  | DD | NA | 3.46 ± 1.39 | 1937.00 ± 1112 | 46.22 ± 26.19 |

**Note:**

DD= Died hamsters

NA=Sample was not available

(*) = P≤0.05

(**) = P≤0.01

(***) =P≤0.001
