# Supplementary material for: Pulmonary haemorrhage as the earliest sign of severe leptospirosis in hamster model challenged with Leptospira interrogans strain HP358
Source: PLoS Negl Trop Dis. 2022 May 18;16(5):e0010409. doi: 10.1371/journal.pntd.0010409 (PMC9116642; doi:10.1371/journal.pntd.0010409)
Supplement: S4 Table — (DOC) [file pntd.0010409.s004.doc]

**S4 Table: Fold gene expression value of anti-inflammatory cytokines**

| **Gene** | **Time (Days)** | **Blood** | **Lungs** | **Liver** | **Kidneys** |
| --- | --- | --- | --- | --- | --- |
| TGF-β1 | Control | 1.71 ± 1.28 | 1.11 ± 0.63 | 1.59 ± 1.33 | 1.05 ± 0.31 |
|  | 1 | 0.57 ± 0.86 | 0.53 ± 0.52 | 0.08 ± 0.83 | 0.53 ± 0.22 |
|  | 3 | 0.97 ± 0.86 | 3.88 ± 0.58 (**) | 0.96 ± 0.71 | 1.16 ± 0.35 |
|  | 4 | 1.39 ± 0.91 | 3.05 ± 0.49(**) | 2.92 ± 1.58 | 1.55 ± 0.42 |
|  | 5 | 1.03 ± 1.03 | 0.28 ± 0.39 | 8.54 ± 1.43 (**) | 3.87 ± 1.19 |
|  | 6 | 1.47 ± 1.01 | 4.23 ± 1.49 | 6.93 ± 1.64 (*) | 5.94 ± 0.34 (***) |
|  | 7 | 0.49 ± 0.83 | 1.58 ± 0.49 | 7.00 ± 1.68 (*) | 9.62 ± 0.69 (***) |
|  | DD | NA | 0.53 ± 0.38 | 9.49 ± 1.22 (**) | 2.12 ± 0.51 |

**Note:**

DD= Died hamsters

NA=Sample was not available

(*) = P≤0.05

(**) = P≤0.01

(***) =P≤0.001
